# Transatlantic differences in the use and outcome of minimally invasive pancreatoduodenectomy: an international multi-registry analysis

**DOI:** 10.1007/s00464-024-11161-7

**Published:** 2024-09-28

**Authors:** Nine de Graaf, Simone Augustinus, Ulrich F. Wellner, Karin Johansen, Bodil Andersson, Joal D. Beane, Bergthor Björnsson, Olivier R. Busch, Catherine H. Davis, Michael Ghadimi, Elizabeth M. Gleeson, Bas Groot Koerkamp, Melissa E. Hogg, Hjalmar C. van Santvoort, Bobby Tingstedt, Waldemar Uhl, Jens Werner, Caroline Williamsson, Herbert J. Zeh, Amer H. Zureikat, Mohammad Abu Hilal, Henry A. Pitt, Marc G. Besselink, Tobias Keck, for the Global Audits on Pancreatic Surgery Group (GAPASURG)

**Affiliations:** 1 Department of Surgery Amsterdam, Amsterdam UMC, location University of Amsterdam, Amsterdam, the Netherlands; 2 Cancer Center Amsterdam, Amsterdam, the Netherlands; 3 Fondazione Poliabulanza Istituto Ospedaliero, Brescia, Italy; 4 DGAV StuDoQ|Pancreas and Clinic of Surgery, UKSH Campus Lübeck, Lübeck, Germany; 5 Department of Surgery in Linköping and Department of Biomedical and Clinical Sciences, Linköping University, Linköping, Sweden; 6 Department of Clinical Sciences Lund, Surgery, Lund University and Skåne University Hospital, Lund, Sweden; 7 Department of Surgery, The Ohio State University, Columbus, OH USA; 8 Department of Surgery, Rutgers Cancer Institute of New Jersey, New Brunswick, NJ USA; 9 Department of Surgery, University Medical Centre Göttingen, Göttingen, the Netherlands; 10 Department of Surgery, Mount Sinai Hospital, New York, NY USA; 11 Department of Surgery, Erasmus MC Cancer Institute, Rotterdam, the Netherlands; 12 Department of Surgery, Northshore University HealthSystem, Evanston, IL USA; 13 Department of Surgery, Regional Academic Cancer Center Utrecht, University Medical Center Utrecht and St. Antonius Hospital Nieuwegein, Utrecht, the Netherlands; 14 Department of Surgery, St. Josef-Hospital Bochum, Bochum, Germany; 15 Department of Surgery, Ludwig-Maximilians-Universität, Munich, Germany; 16 Department of Surgery, University of Texas Southwestern Medical Center, Dallas, TX USA; 17 Department of Surgery, University of Pittsburgh Medical Center, Pittsburgh, USA; 18 Department of Surgery, UKSH Campus Lübeck, Ratzeburger Allee 160, 23562 Lübeck, Germany; 19 Department of Surgery Amsterdam, Amsterdam UMC, Location University of Amsterdam, De Boelelaan 1117 (ZH-7F), 1081 HV Amsterdam, the Netherlands

**Keywords:** Minimally invasive surgery, pancreatoduodenectomy, Whipple, Pancreatic cancer, Robotic, Robot-assisted, Laparoscopy

## Abstract

**Background:**

Minimally invasive pancreatoduodenectomy (MIPD) has emerged as an alternative to open pancreatoduodenectomy (OPD). However, the extent of variation in the use and outcomes of MIPD in relation to OPD among countries is unclear as international studies using registry data are lacking. This study aimed to investigate the use, patient selection, and outcomes of MIPD and OPD in four transatlantic audits for pancreatic surgery.

**Methods:**

A post hoc comparative analysis including consecutive patients after MIPD and OPD from four nationwide and multicenter pancreatic surgery audits from North America, Germany, the Netherlands, and Sweden (2014–2020). Patient factors related to MIPD were identified using multivariable logistic regression. Outcome analyses excluded the Swedish audit because < 100 MIPD were performed during the studied period.

**Results:**

Overall, 44,076 patients who underwent pancreatoduodenectomy were included (29,107 North America, 7586 Germany, 4970 the Netherlands, and 2413 Sweden), including 3328 MIPD procedures (8%). The use of MIPD varied widely among countries (absolute largest difference [ALD] 17%, *p* < 0.001): 7% North America, 4% Germany, 17% the Netherlands, and 0.1% Sweden. Over time, the use of MIPD increased in North America and the Netherlands (*p* < 0.001), mostly driven by robotic MIPD, but not in Germany (*p* = 0.297). Patient factors predicting the use of MIPD included country, later year of operation, better performance status, high POPF-risk score, no vascular resection, and non-malignant indication. Conversion rates were higher in laparoscopic MIPD (range 28–45%), compared to robotic MIPD (range 9–37%). In-hospital/30-day mortality differed among North America, Germany, and the Netherlands; MIPD (2%, 7%, 4%; ALD 5%, *p* < 0.001) and OPD (2%, 5%, 3%; ALD 3%, *p* < 0.001), similar to major morbidity; MIPD (25%, 42%, 38%, ALD 17%, *p* < 0.001) and OPD (25%, 31%, 30%, ALD 6%, *p* < 0.001), respectively.

**Conclusions:**

Considerable differences were found in the use and outcome, including conversion and mortality rates, of MIPD and OPD among four transatlantic audits for pancreatic surgery. Our findings highlight the need for international collaboration to optimize treatment standards and patient outcome.

**Supplementary Information:**

The online version contains supplementary material available at 10.1007/s00464-024-11161-7.

Over the past decade, a growing emphasis has been placed on outcome assessment in surgery, aiming to monitor safety and improve of quality of care [1]. Particularly in pancreatic surgery, recording of outcomes is of great importance, as outcomes vary widely among centers and surgeons [2, 3]. As a result, several countries have established prospective audits on pancreatic surgery, enabling both quality control and clinical research [4–6].

For the introduction of novel surgical techniques, such as minimally invasive pancreatoduodenectomy (MIPD), auditing facilitates a controlled implementation in clinical practice. MIPD aims to improve patient outcome and enhance postoperative recovery compared to open pancreatoduodenectomy (OPD), with promising results reported by expert centers [7–12]. However, implementing MIPD on a national scale requires overcoming the learning curve in a large number of centers which may not all have sufficient annual surgical volume. To address this, national and international audits have been recommended for monitoring MIPD implementation in clinical practice [13, 14]. However, a comprehensive analysis of the use and outcome of MIPD among different countries based on registry data is so far lacking.

The Global Audits on Pancreatic Surgery Group (GAPASURG) includes the audits on pancreatic surgery from North America, the Netherlands, Germany, and Sweden [15]. GAPASURG allows for a detailed assessment on the use and outcome of MIPD among the four audits. Differences could be explained by different implementation strategies and patient selection or center characteristics including low annual volume [13].

The GAPASURG collaboration offers a unique opportunity to investigate potential international differences in use and outcome of MIPD and open pancreatoduodenectomy (OPD). Therefore, the aim in this study was to provide an overview of the patient selection, use, and outcome of MIPD in these four transatlantic audits, in relation to OPD.

## Methods

### Study design

This study is a post hoc analysis including all consecutive adults after pancreatoduodenectomy for all indications from the four audits collaborating within the Global Audits on Pancreatic Surgery Group (2014–2020) [15]. The audits include the United States and Canada (American College of Surgeons National Surgical Quality Improvement Program [NSQIP]) [4]: multicenter, 170 centers in 2020); Germany (Deutsche Gesellschaft für Allgemein- und Viszeral Chirurgie- Studien-, Dokumentations- und Qualitätszentrum [DGAV StudoQ |Pancreas] [6]: multicenter, 67 centers in 2020); the Netherlands (Dutch Pancreatic Cancer Audit [DPCA] [5, 16]: nationwide, 17 centers in 2020) and Sweden (Swedish National Pancreatic and Periampullary Cancer Registry [17]: nationwide, 6 centers in 2020). Among these, the North American and German audits are multicenter (participation is voluntary) and the Dutch and Swedish audits are nationwide (participation is mandatory for all centers). Ethical approval was obtained for all audits individually, and no ethical committee review was required for this study due to the use of anonymous data. The study was reported in accordance with the STROBE guidelines [18].

### Outcomes

The main outcome of interest was the use of MIPD in the four audits. Secondary outcomes included (1) differences in characteristics of patients undergoing MIPD and OPD; (2) predictors of performing MIPD, (3) differences in outcomes after MIPD and OPD, (4) differences in outcomes after MIPD and OPD. If a registry included < 100 patients undergoing MIPD, indicating that this country is in the early implementation phase, these patients were excluded from the outcome analysis. Differences were assessed both within and between audits and as trends in time.

### Data collection

The baseline variables, surgical characteristics, pathological variables, and postoperative outcomes characteristics were derived from the four audits: NSQIP, StudoQ, DPCA, and the Swedish registry. In all four registries, information on patient and tumor characteristics, diagnosis and treatment outcomes is routinely extracted from the medical records by independent trained administrators (e.g. research nurses). NSQIP collect data for 30 days after surgery. StuDoQ, DPCA and the Swedish registry collect data during the complete primary hospital stay or up to 30 days after surgery in case of earlier discharge. Baseline variables included: country, age, sex, body mass index (BMI), American Society of Anesthesiologist (ASA) classification, performance status (World Health organization [WHO] or ECOG), neo-adjuvant chemo(radio)therapy. Surgical characteristics included: operation year, type of pancreatoduodenectomy (pylorus preserving PD, pylorus resecting PD/classic Whipple), operative approach (open, laparoscopic, laparoscopic including conversion, robotic, robotic including conversion), vascular resection. Pathological variables included: histopathological diagnosis, and tumor stage. Postoperative outcomes included pancreatic surgery specific morbidity defined according to the International Study Group for Pancreatic Surgery (ISGPS), only including clinically relevant complications (grade B/C): postoperative pancreatic fistula (POPF) [19], bile leak, delayed gastric emptying (DGE) [20], and post-pancreatectomy hemorrhage (PPH) [21]. Other endpoints included: surgical site infection (SSI) [22], organ failure, intensive care unit (ICU) admission, morbidity according to Clavien–Dindo classification [23], major morbidity (defined as Clavien–Dindo grade 3 or higher) reoperation, date of discharge, readmission, and in-hospital/30-day mortality. Anonymized center data was only available for StudoQ, DPCA and the Swedish registry. For this reason volume analyses to identify the centers’ learning phases are limited to these registries. Cutoffs for the feasibility, proficiency, and mastery learning phases were set at 15, 62, and 84 MIPD [24].

### Definitions

Operative approach was defined as open procedure (laparotomy), laparoscopic procedure with conversion, laparoscopic procedure without conversion, robotic procedure with conversion, robotic procedure without conversion, or other procedures. Other approaches include hybrid procedures performed within the North American registry. MIPD included both laparoscopic (with and without conversion), and robotic (with and without conversion) procedures. Other approaches were included in the total cohort, but excluded from outcome analysis comparing MIPD and OPD, and identification of predictors for MIPD. Patients were classified as high risk for POPF when the pancreatic duct size was ≤ 3 mm or BMI > 30 kg/m^2^, and as low/moderate risk when the duct size was either > 3 mm or BMI was ≤ 30 kg/m^2^. Safety outcomes were defined as in-hospital/30-day mortality and severe complications (Clavien–Dindo grade ≥ 3).

Differences in parameters due to the various metric systems were resolved by converting the data, ounces were converted to kilograms and inches into meters. Several variables were recategorized so that data could be combined. For example, ECOG performance status were recategorized to match functional health status of independent (ECOG 0 or 1) or partially dependent (ECOG 2 or 3), and totally dependent (ECOG 4). Moreover, vascular resection was used as a surrogate to determine pre-operative vascular involvement, which was not available within all audits. Ideal outcome was defined as the absence of: (1) mortality, (2) Clavien–Dindo ≥ 3 morbidity, (3) postoperative pancreatic fistula (grade B/C; POPF), (4) reoperation, (5) hospital stay > 75th percentile, and (6) readmission. All outcomes were measured during primary admission and/or 30 days postoperatively.

### Statistical analyses

Descriptive statistics are used to assess baseline characteristics and primary and secondary outcomes. Results are reported as proportions for binary or categorical variables, and as mean with standard deviation (SD) or as median with interquartile range (IQR) for continuous variables as appropriate in case of normally or not-normally distributed data, respectively. First, normally distributed data were compared using a Students-*t*-test, categorical data using the chi-square test, and non-normally distributed data using the Mann Whitney *U* test. Relative and absolute largest differences (RLD, ALD) were used to report differences among the groups, because even minimal differences were found statistically significant with this large dataset (> 40,000 cases).

Second, linear regression analysis was performed to investigate the increase/decrease of MIS over time. Third, multivariable logistic regression analyses were performed to investigate the independent predictors for performing MIS. Values included in this analysis were: age, sex, country, BMI, comorbidities (diabetes, chronic obstructive pulmonary disease (COPD), cardiac heart failure, dialysis), performance status, ASA score, pre-operative biliary drainage, operation year, risk classification for POPF, vascular resection, and histological diagnosis. All variables with a *p*-value < 0.2 in univariable analysis were added to the multivariable regression model. Variables were excluded through backward selection until only statistically significant variables were left in the final multivariable model. Fourth, the association of operative approach (MIPD or OPD) with postoperative outcomes (i.e. Clavien–Dindo ≥ 3 morbidity, POPF, and in-hospital/30-day mortality), also was investigated using multivariable logistic regression models. First, univariable analysis was performed investigating association of operative approach and the three postoperative outcomes. When *p*-value was < 0.2, multivariable logistics regression was performed correcting for all the independent predictors for performing MIPD (in at least one registry) identified in the previous analysis.

Patients were analyzed according to the intention-to-treat principle, meaning that patients who underwent a MIPD with conversion to OPD were analyzed in the MIPD group. Missing values on baseline characteristics were imputed by multiple imputation techniques using ten dummy sets and used in logistic regression analysis. All reported *p* values were based on a 2-sided test, and a *p* value of < 0.05 was considered statistically significant. All calculations were be performed with RStudio (version 4.0.3).

## Results

Overall, 44,076 patients after pancreatoduodenectomy were included: 29,107 (66%) from North America, 7586 (17%) Germany, 4970 (11%) the Netherlands, and 2413 (5%) Sweden. Baseline characteristics are presented in Table 1. In total, 3328 (8%) patients underwent MIPD. Of all included patients, 46% were female, and the median age was 67 years (IQR: 59–74). The percentage of patients operated for pancreatic ductal adenocarcinoma (PDAC) was 55% in the total cohort, and in differed among countries: 59% in North America, 51% in Germany, 42% in the Netherlands, and 51% in Sweden (ALD 17%, RLD 1.4, *p* < 0.001). The use of MIPD varied between 7% in North America, 4% in Germany, 17% in the Netherlands, and 0.1% in Sweden (ALD 17%, RLD 170, *p* < 0.001). Because Sweden included < 100 patients who underwent MIPD, these patients were excluded from further analysis.

**Table 1:** Baseline characteristics and operative outcomes of patients after pancreatoduodenectomy in four transatlantic audits

	North America (*n* = 29,107)	Germany (*n* = 7586)	The Netherlands (*n* = 4970)	Sweden (*n* = 2413)	Total (*n* = 44,076)
Age, median (IQR)	66.0 (58.0–73.0)	69.0 (60.0–76.0)	68.0 (61.0–74.0)	70.0 (63.2–75.0)	67.0 (59.0–74.0)
Female	13,534 (46%)	3267 (43%)	2210 (45%)	1152 (48%)	20,163 (46%)
BMI, median (IQR)	26.5 (23.3–30.4)	24.9 (22.5–27.8)	24.8 (22.4–27.6)	24.9 (22.5–27.8)	25.8 (23.0–29.5)
Diabetes	7714 (27%)	1928 (25%)	1009 (25%)	497 (21%)	11,148 (25%)
COPD	1179 (4%)	371 (5%)	549 (14%)	130 (6%)	2229 (5%)
Heart failure	114 (0.4%)	936 (13%)	123 (3%)	640 (27%)	1813 (4%)
Dialysis	92 (0.3%)	23 (0.3%)	163 (4%)	9 (4%)	287 (1%)
Performance status					
Independent	28,825 (99%)	7199 (95%)	3905 (91%)	2204 (93%)	42,133 (97%)
Partially dependent	226 (1%)	310 (4%)	373 (9%)	157 (7%)	1066 (3%)
Fully dependent	20 (< 0.1%)	57 (1%)	1 (< 0.1%)	1 (< 0.1%)	79 (0.2%)
ASA score ≥ 3	23,113 (79%)	3894 (51%)	1355 (29%)	669 (28%)	29,031 (66%)
Pre-operative biliary drainage					
No	12,783 (46%)	4740 (63%)	2204 (46%)	1009 (42%)	20,736 (49%)
Yes – ERCP	14,191 (51%)	2831 (37%)	2330 (49%)	1384 (58%)	20,736 (49%)
Yes – PTCD^b^	859 (3%)	NR	226 (5%)	NR	1085 (3%)^b^
Neoadjuvant chemotherapy^a^	5621 (34%)	230 (6%)	342 (16%)	68 (6%)	6261 (26%)
Histological diagnosis					
Pancreatic adenocarcinoma	16,795 (59%)	3813 (51%)	2064 (42%)	1133 (51%)	23,805 (55%)
Ampullary carcinoma	843 (3%)	603 (8%)	634 (13%)	247 (11%)	2327 (5%)
Distal cholangiocarcinoma	2189 (8%)	631 (8%)	656 (13%)	295 (13%)	3771 (9%)
Duodenal carcinoma	879 (3%)	199 (3%)	313 (6%)	157 (7%)	1548 (4%)
Neuroendocrine tumor	1793 (6%)	279 (4%)	236 (5%)	42 (2%)	2350 (5%)
IPMN	1779 (6%)	434 (6%)	364 (7%)	21 (1%)	2598 (6%)
MCN/serous cystadenoma	503 (2%)	126 (2%)	41 (1%)	0 (0%)	670 (2%)
Chronic pancreatitis	1079 (4%)	740 (10%)	139 (3%)	67 (3%)	2025 (5%)
SPN	127 (0.4%)	34 (1%)	20 (0.4%)	0 (0%)	181 (0.4%)
Intestinal adenoma	0 (0%)	0 (0%)	130 (3%)	61 (3%)	191 (0.4%)
Other	1630 (9%)	663 (9%)	312 (6%)	194 (9%)	3799 (9%)
Type of PD					
Pylorus preserving PD	10,456 (36%)	5433 (72%)	2546 (51%)	581 (24%)	19,016 (43%)
Pylorus resecting PD/classic Whipple	18,651 (64%)	2153 (28%)	2424 (49%)	1832 (76%)	25,060 (57%)
MIPD	2143 (8%)	303 (4%)	839 (17%)	21 (0.1%)	3285 (8%)
Number of centers performing MIPD/total number of centers	NA/170	3/67	8/17	1/6	NA/260
Vascular resection^c^					
No	23,505 (82%)	6659 (88%)	4141 (84%)	1968 (82%)	36,273 (83%)
Vein	3697 (12%)	900 (12%)	696 (14%)	426 (18%)	5719 (13%)
Artery	581 (2%)	21 (0.3%)	54 (1%)	8 (0.3%)	664 (2%)
Vein and artery	961 (3%)	6 (< 0.1%)	20 (0.4%)	8 (0.3%)	995 (2%)

Numbers are depicted as *N* (%) unless indicated otherwise. Details regarding numbers of patients with missing values can be found in Supplementary Table 13

*MIPD* minimally invasive pancreatoduodenectomy, *OPD* open pancreatoduodenectomy, *NR* not registered, *ALD* absolute largest difference, *RLD* relative largest difference

^a^Used as a surrogate for pre-operative vascular involvement

^b^Comparing patients undergoing MIS surgery among the GAPASURG countries

^c^Only comparing vascular resection (yes or no), as groups become too small to compare

### Trend in use of MIPD

**Fig. 1:**
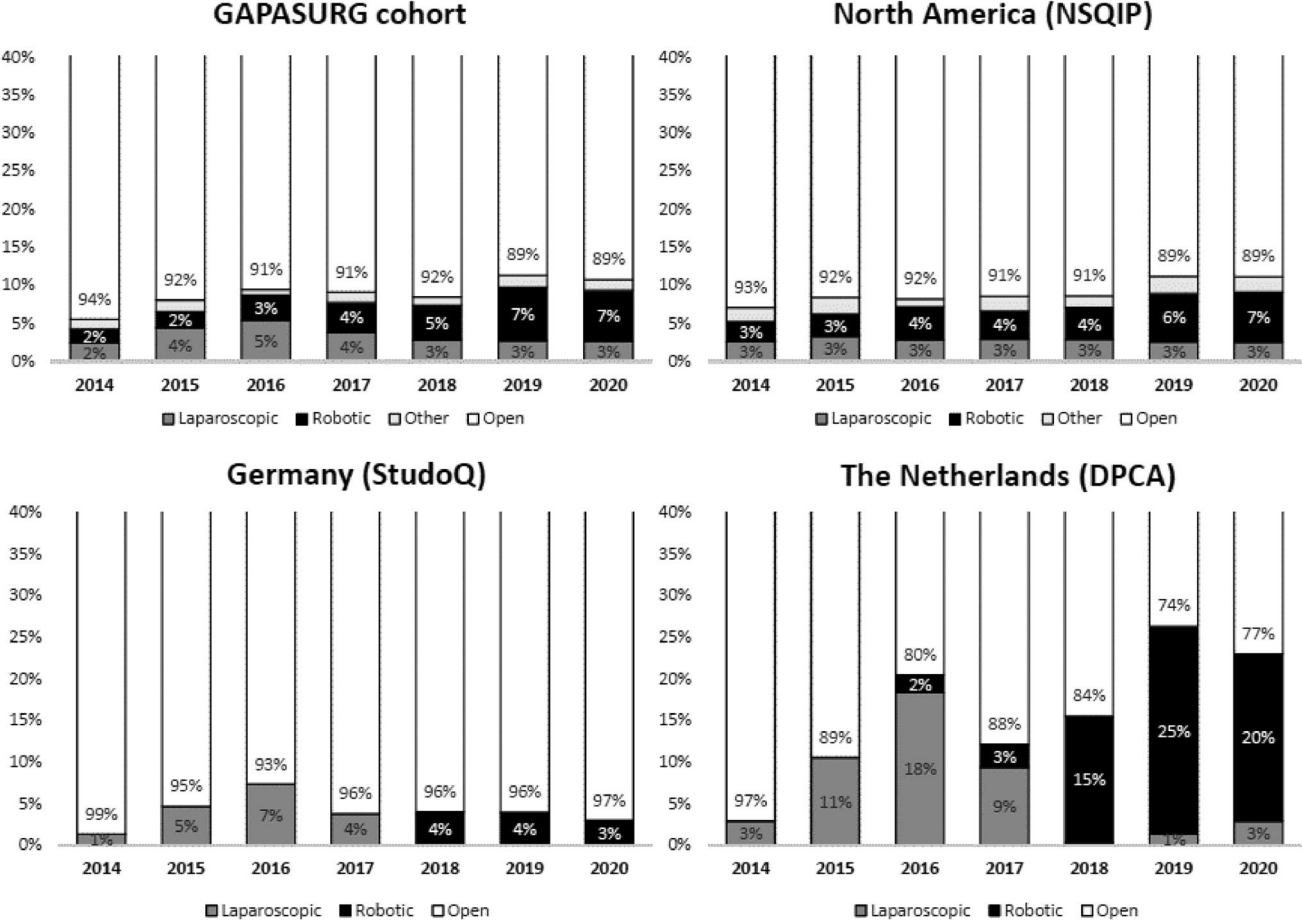
Trends in the use of MIPD, stratified for laparoscopic and robotic, in three transatlantic audits of pancreatic surgery

In the first study year (2014), in Germany and the Netherlands only laparoscopic MIPD was performed, while in North America both laparoscopic and robotic MIPD were performed (Fig. 1). The implementation of robotic MIPD started in the Netherlands in 2016 and in Germany in 2018. In the total cohort, the use of MIPD increased over time (*p* < 0.001). Per country, the use of MIPD increased over time in both North America and the Netherlands, (both: *p* < 0.001), both mostly driven by robotic MIPD, but not in Germany (*p* = 0.297). The median total MIPD volume per center contributing data was 47 (range 6–122) in the Netherlands, 33 (range 10–143) in Germany and 21 in Sweden. During the study period, 3/11 Dutch centers passed the mastery learning phase, 1/11 centers passed the proficiency learning phase and 7/11 centers passed the feasibility learning phase. 1/3 German centers passed the mastery learning phase, 1/3 centers passed the proficiency learning phase and 1/3 center was still in the early learning phase. The single Swedish MIPD center passed the feasibility learning phase. Conversion rates were higher during laparoscopic MIPD (range 28–45%), compared to robotic MIPD (range 9–37%) (Suppl Table 1). Between North America, Germany, and the Netherlands, the lowest conversion rate for laparoscopic MIPD (45%, 35%, and 28%; *p* < 0.001) and robotic MIPD (15%, 37%, and 9%; *p* < 0.001) was reported in the Netherlands.

### Patient characteristics

Demographics of patients undergoing MIPD differed among countries (Table 2). The difference among countries was highest (ALD more than 10%) for BMI ≥ 30 kg/m^2^, COPD, ASA score, and biliary drainage. Patients who underwent MIPD in North America more often had a BMI ≥ 30 (ADL: 16%), and ASA score ≥ 3 (ALD: 36%). Patients undergoing MIPD in the Netherlands had more often COPD (ALD: 12%). Patients undergoing MIPD in Germany more often underwent biliary drainage (ALD: 27%). Most of these differences, except biliary drainage, were also evident in patients undergoing OPD.

**Table 2:** Characteristics of patients after MIPD and OPD in three transatlantic audits

	North America		*p*-value	Germany		*p*-value	The Netherlands		*p*-value	MIS GAPASURG^a^		
	MIPD (*n* = 2143)	OPD (*n* = 26,431)		MIPD (*n* = 303)	OPD (*n* = 7264)		MIPD (*n* = 839)	OPD (*n* = 4041)		ALD	RLD	*p*-value
Age, median (IQR)	66.0 (58.0–73.0)	66.0 (58.0–73.0)	0.237	69.0 (58.5–76.0)	69.0 (60.0–76.0)	0.353	69.0 (61.0–75.0)	68.0 (60.0–74.0)	**0.010**	3.0	1.0	** < 0.001**
Female	1011 (47%)	12,277 (46%)	0.516	134 (44%)	3123 (43%)	0.676	361 (43%)	1809 (45%)	0.380	4%	1.1	0.114
BMI, median (IQR)	26.9 (23.7–30.9) *5*	26.5 (23.3–30.3) *148*	** < 0.001**	25.5 (23.1–28.3) *0*	24.9 (22.5–27.8) *25*	**0.030**	25.1 (22.6–27.7) *25*	24.7 (22.3–27.5) *181*	**0.065**	1.8	1.1	** < 0.001**
BMI ≥ 30 kg/m^2^	610 (29%)	6962 (26%)	**0.040**	46 (15%)	1048 (14%)	0.733	106 (13%)	501 (13%)	0.865	16%	2.2	** < 0.001**
Diabetes	541 (25%)	7031 (27%)	0.171	65 (21%)	1859 (26%)	0.104	172 (24%)	820 (25%)	0.307	4%	1.2	** < 0.001**
COPD	88 (4%)	1079 (4%)	0.957	15 (5%)	356 (5%)	0.970	114 (16%)	424 (13%)	0.075	12%	4.0	0.288
Cardiac heart failure	8 (0.4%)	106 (0.4%)	0.845	16 (5%)	918 (13%)	** < 0.001**	28 (4%)	94 (3%)	0.191	4.6%	12.5	** < 0.001**
Dialysis	6 (0.3%)	83 (0.3%)	0.786	1 (0.3%)	22 (0.3%)	0.610	36 (5%)	126 (4%)	0.203	4.7%	4.7	** < 0.001**
Performance status												
Independent	2129 (99%)	26,168 (99%)	0.427	298 (98%)	6889 (95%)	**0.020**	672 (93%)	3150 (91%)	**0.046**	6%	1.1	** < 0.001**
Partially depended	13 (1%)	209 (1%)		4 (1%)	305 (4%)		48 (7%)	322 (9%)		6%	7.0	
Fully dependent	0 (0%)	20 (< 0.1%)		1 (0.3%)	56 (1%)		0 (0%)	1 (< 0.1%)		1%	NA	
ASA score ≥ 3	1637 (77%)	21,071 (80%)	** < 0.001**	167 (55%)	3716 (51%)	0.178	234 (29%)	1102 (28%)	0.610	48%	2.7	** < 0.001**
Biliary drainage												
No	999 (48%)	11,555 (46%)	**0.012**	216 (71%)	4519 (62%)	**0.002**	354 (44%)	1814 (47%)	0.150	27%	1.6	** < 0.001**
Yes – ERCP	1029 (50%)	12,919 (51%)		88 (29%)	2739 (38%)		423 (52%)	1859 (48%)				
Yes – PTCD	46 (2%)	800 (3%)		NR	NR		35 (4%)	186 (5%)				
High risk for POPF	1591 (79%)	18,429 (76%)	**0.001**	221 (85%)	4930 (80%)	0.057	575 (79%)	2423 (73%)	** < 0.001**	6%	1.1	0.108
Vascular resection^b^												
No	1846 (87%)	21,231 (81%)	** < 0.001**	266 (88%)	6374 (88%)	** < 0.001**	769 (92%)	3303 (83%)	** < 0.001**	5%	1.1	** < 0.001**^**c**^
Vein	180 (9%)	3464 (13%)		36 (12%)	864 (12%)		58 (7%)	626 (16%)		5%	1.7	
Artery	42 (2%)	524 (2%)		1 (0.3%)	20 (0.3%)		5 (1%)	47 (1%)		2%	6.7	
Vein and artery	48 (2%)	887 (3%)		0 (0%)	6 (< 0.1%)		2 (0.2%)	18 (1%)		2%	NA	
Malignant disease	1442 (69%)	18,909 (73%)	** < 0.001**	178 (59%)	5055 (70%)	** < 0.001**	585 (74%)	3013 (77%)	**0.023**	15%	1.3	** < 0.001**

Numbers are depicted as *N* (%) unless indicated otherwise

*MIPD* minimally invasive pancreatoduodenectomy, *OPD* open pancreatoduodenectomy, *NR* not registered, *ALD* absolute largest difference, *RLD* relative largest difference

^a^Comparing patients undergoing MIS surgery among the GAPASUG countries

^b^Used as a surrogate for pre-operative vascular involvement

^c^Only comparing vascular resection (yes or no), as groups become too small to compare. Bold values indicate the statistical significance. Details regarding numbers of patients with missing values can be found in Supplementary Table 14

Independent predictors for undergoing MIPD in the total cohort, were country (the Netherlands), no heart failure, better performance status, later year of operation, high risk category for POPF, and no vascular resection (Suppl Table 2). Patients with ampullary cancer, neuroendocrine tumors and IPMN had a higher chance of receiving MIPD compared to patients who underwent surgery for PDAC and chronic pancreatitis.

Independent predictors for undergoing MIPD were also assessed per individual audit (Table 3). In all audits, patients with a malignant indication for surgery less often underwent MIPD. In North America, MIPD was additionally independently associated with higher BMI, lower ASA score, no pre-operative percutaneous transhepatic biliary drainage, later operation year, and no vascular resection. Within Germany, MIPD was independently associated with no cardiac heart failure, better performance status, higher ASA score, and no biliary drainage. In the Netherlands, MIPD was independently associated with higher age, biliary drainage, later operation year, high risk category for POPF, and no vascular resection.

**Table 3:** Predictors for the use of MIPD in three transatlantic audits

	North America (*n* = 2143)				Germany (*n* = 303)				The Netherlands (*n* = 839)			
	Univariable analysis OR (95 CI)	*p*-value^a^	Multivariable analysis OR (95 CI)	*p*-value^b^	Univariable analysis OR (95 CI)	*p*-value^a^	Multivariable analysis OR (95 CI)	*p*-value^b^	Univariable analysis OR (95 CI)	*p*-value^a^	Multivariable analysis OR (95 CI)	*p*-value^b^
Age	0.99 (0.99–1.00)	**0.145**			0.99 (0.98–1.00)	0.219			1.01 (1.00–1.02)	**0.008**	1.01 (1.00–1.02)	**0.007**
Female	1.02 (0.94–1.12)	0.516			1.05 (0.83–1.32)	0.677			0.93 (0.80–1.08)	0.362		
BMI	1.01 (1.01–1.02)	** < 0.001**	1.01 (1.00–1.02)	**0.002**	1.01 (0.99–1.02)	0.367			0.99 (0.99–1.00)	0.645		
Diabetes	0.93 (0.84–1.03)	**0.171**			0.79 (0.60–1.05)	**0.105**			0.92 (0.76–1.12)	0.409		
COPD	1.01 (0.81–1.26)	0.957			1.01 (0.59–1.71)	0.972			1.15 (0.93–1.43)	0.204		
Cardiac heart failure	0.93 (0.45–1.91)	0.844			0.38 (0.23–0.64)	** < 0.001**	0.39 (0.24–0.65)	** < 0.001**	1.25 (0.81–1.92)	0.314		
Dialysis	0.89 (0.39–2.04)	0.786			1.09 (0.15–8.11)	0.932			1.25 (0.86–1.84)	0.248		
Performance status												
Independent	Reference				Reference		Reference		Reference			
Partially dependent	0.76 (0.43–1.34)	0.345			0.30 (0.11–0.82)	**0.018**	0.33 (0.12–0.89)	**0.029**	0.74 (5.45–1.01)	**0.062**		
Fully dependent	Infinite	0.926			0.41 (0.06–1.96)	0.377	0.41 (0.06–3.01)	0.384	Infinite	0.959		
ASA score ≥ 3	0.83 (0.75–0.92)	** < 0.001**	0.83 (0.74–0.92)	** < 0.001**	1.18 (0.93–1.48)	**0.178**	1.35 (1.07–1.71)	**0.011**	1.06 (0.89–1.25)	0.529		
Biliary drainage												
No	Reference		Reference		Reference		Reference		Reference		Reference	
Yes – ERCP	0.93 (0.85–1.01)	**0.102**	0.99 (0.89–1.09)	0.868	0.68 (0.52–0.89)	**0.002**	0.75 (0.58–0.97)	**0.031**	1.15 (0.99–1.34)	**0.070**	1.21 (1.03–1.44)	**0.024**
Yes – PTCD	0.67 (0.49–0.91)	**0.010**	0.71 (0.52–0.97)	**0.034**	NR	NA			0.97 (0.66–1.43)	0.883	1.03 (0.69–1.54)	0.871
Operation year	1.09 (1.07–1.12)	** < 0.001**	1.10 (1.08–1.13)	** < 0.001**	0.97 (0.91–1.03)	0.297			1.25 (1.20–1.30)	** < 0.001**	1.25 (1.20–1.30)	** < 0.001**
Low risk for POPF	0.93 (0.74–0.94)	**0.002**			0.77 (0.54–1.09)	**0.149**			0.74 (0.59–0.92)	**0.009**	0.75 (0.59–0.95)	**0.022**
Vascular resection	0.64 (0.56–0.73)	** < 0.001**	0.65 (0.57–0.75)	** < 0.001**	0.99 (0.70–1.42)	0.982			0.40 (0.31–0.53)	** < 0.001**	0.39 (0.30–0.52)	** < 0.001**
Malignant diagnosis	0.82 (0.75–0.91)	** < 0.001**	0.87 (0.79–0.97)	**0.012**	0.61 (0.48–0.77)	** < 0.001**	0.66 (0.51–0.84)	** < 0.001**	0.82 (0.69–0.98)	**0.029**	0.79 (0.65–0.96)	**0.018**

*NR* not registered, *NA* not applicable, *CI* confidence interval, *BMI* body mass index (kg/m^2^), *COPD* chronic obstructive pulmonary disease, *ASA* American Society of Anesthesiologists physical status classification system, *ERCP* endoscopic retrograde cholangio- and pancreaticography, *PTCD* percutaneous transhepatic cholangiodrainage, *POPF* postoperative pancreatic fistula

^a^Bold numbers indicate a value < 0.2 and thereby added into multivariable analysis

^b^Bold numbers indicate statistical significance. Details regarding numbers of patients with missing values can be found in Supplementary Table 15

### Outcomes

#### MIPD and OPD

Outcomes after MIPD and OPD are presented in Table 4. Within North America, none of the safety outcomes differed between patients undergoing MIPD and OPD (Table 4). In Germany and the Netherlands, patients undergoing MIPD had significantly worse safety outcomes, except for mortality, as compared to OPD. The rate of major morbidity after MIPD compared to OPD was significantly higher in Germany (42 vs 31%) and the Netherlands (38 vs 30%), compared to no significant difference seen in North America (25 vs 25%). In Germany, patients who underwent MIPD had more bile leakage (9 vs 5%) and PPH (16 vs 9%), compared to OPD, but the rates of POPF (17 vs 14%) and DGE (11 vs 10%) did not significantly differ. In the Netherlands, all pancreatic surgery specific morbidity was higher for patients undergoing MIPD, compared to OPD: POPF (24 vs 15%), bile leakage (9 vs 5%), DGE (9 vs 15%) and PPH (13 vs 7%). Surgical site infection was lower for patients undergoing MIPD in North America (7 vs 10%) and the Netherlands (7 vs 11%), and not different in Germany (13 vs 12%). In North America, fewer patients after MIPD had a prolonged length of stay, although a higher readmission rate was found as compared to OPD. In Germany and the Netherlands, length of hospital stay and the readmission rate did not differ between MIPD and OPD. In North America, the rate of patients achieving Ideal Outcome did not differ between MIPD and OPD, while in Germany and the Netherlands this rate was lower after MIPD.

**Table 4:** Outcomes after MIPD and OPD in GAPASURG and three transatlantic audits of pancreatic surgery

	North America		*p*-value	Germany		*p*-value	The Netherlands		*p*-value	MIS GAPASURG^a^		
	MIPD (*n* = 2143)	OPD (*n* = 26,431)		MIPD (*n* = 303)	OPD (*n* = 7264)		MIPD (*n* = 839)	OPD (*n* = 4041)		ALD	RLD	*p*-value
Safety outcomes												
Clavien–Dindo ≥ 3	532 (25%)	6533 (25%)	0.911	127 (42%)	2265 (31%)	** < 0.001**	316 (38%)	1191 (30%)	** < 0.001**	17%	1.7	** < 0.001**
Radiologic intervention	299 (14%)	3629 (19%)	0.770	NR	NR	NA	236 (29%)	769 (20%)	** < 0.001**	15%	2.1	** < 0.001**
Reoperation	145 (7%)	1459 (6%)	**0.016**	77 (25%)	1198 (17%)	** < 0.001**	87 (11%)	306 (8%)	**0.009**	18%	3.6	** < 0.001**
Unplanned ICU admission	NR	NR	NA	46 (15%)	776 (11%)	**0.014**	115 (14%)	412 (10%)	**0.003**	1%	1.1	0.566
In-hospital/30-day mortality	35 (2%)	390 (2%)	0.563	20 (7%)	368 (5%)	0.237	29 (4%)	132 (3%)	0.780	5%	7.2	** < 0.001**
Ideal outcome not achieved	944 (44%)	11,849 (45%)	0.524	162 (54%)	3323 (46%)	**0.007**	431 (53%)	1858 (47%)	**0.006**	13%	1.3	** < 0.001**
Other morbidity												
POPF grade B/C	212 (10%)	2685 (10%)	0.702	52 (17%)	1014 (14%)	0.119	201 (24%)	602 (15%)	** < 0.001**	14%	2.4	** < 0.001**
Bile leakage grade B/C	NR	NR	NA	27 (9%)	378 (5%)	**0.005**	74 (9%)	197 (5%)	** < 0.001**	0%	NA	0.989
DGE grade B/C	358 (17%)	4407 (17%)	0.986	33 (11%)	731 (10%)	0.646	74 (9%)	751 (5%)	** < 0.001**	2%	1.2	** < 0.001**
PPH grade B/C	NR	NR	NA	49 (16%)	665 (9%)	** < 0.001**	106 (13%)	295 (7%)	** < 0.001**	3%	1.2	0.142
Pneumonia	66 (3%)	1059 (4%)	**0.034**	33 (11%)	434 (6%)	** < 0.001**	45 (6%)	158 (5%)	0.242	8%	3.7	** < 0.001**
Surgical site infection	158 (7%)	2562 (10%)	** < 0.001**	39 (13%)	834 (12%)	0.424	51 (7%)	338 (11%)	**0.002**	6%	1.9	**0.001**
Other outcomes												
LOS < 75 percentile	411 (20%)	6467 (25%)	** < 0.001**	77 (26%)	1738 (24%)	0.498	201 (24%)	936 (23%)	0.577	14%	2.2	** < 0.001**
Readmission	427 (20%)	4462 (17%)	** < 0.001**	33 (11%)	624 (9%)	0.170	149 (19%)	678 (18%)	0.451	9%	1.8	** < 0.001**

Bold numbers indicate statistical significance

^a^ Comparing patients undergoing MIS surgery among the GAPASUG countries

*ICU* intensive care unit, *POPF* postoperative pancreatic fistula, *DGE* delayed gastric emptying, *PPH* post-pancreatectomy hemorrhage, *LOS* length of hospital stay in days

In multivariable analysis, MIPD was independently associated with higher rate of major morbidity, POPF, and failure to achieve Ideal Outcome, but not with mortality (Suppl Tables 3–9). Within North America, no association was observed between operative approach and the studied postoperative outcomes. In Germany, MIPD was associated with increased risk of major morbidity and failure to achieve Ideal Outcome. These findings also were the same in the Netherlands, with an additional association with POPF.

#### Trends in safety outcomes over time

**Fig. 2:**
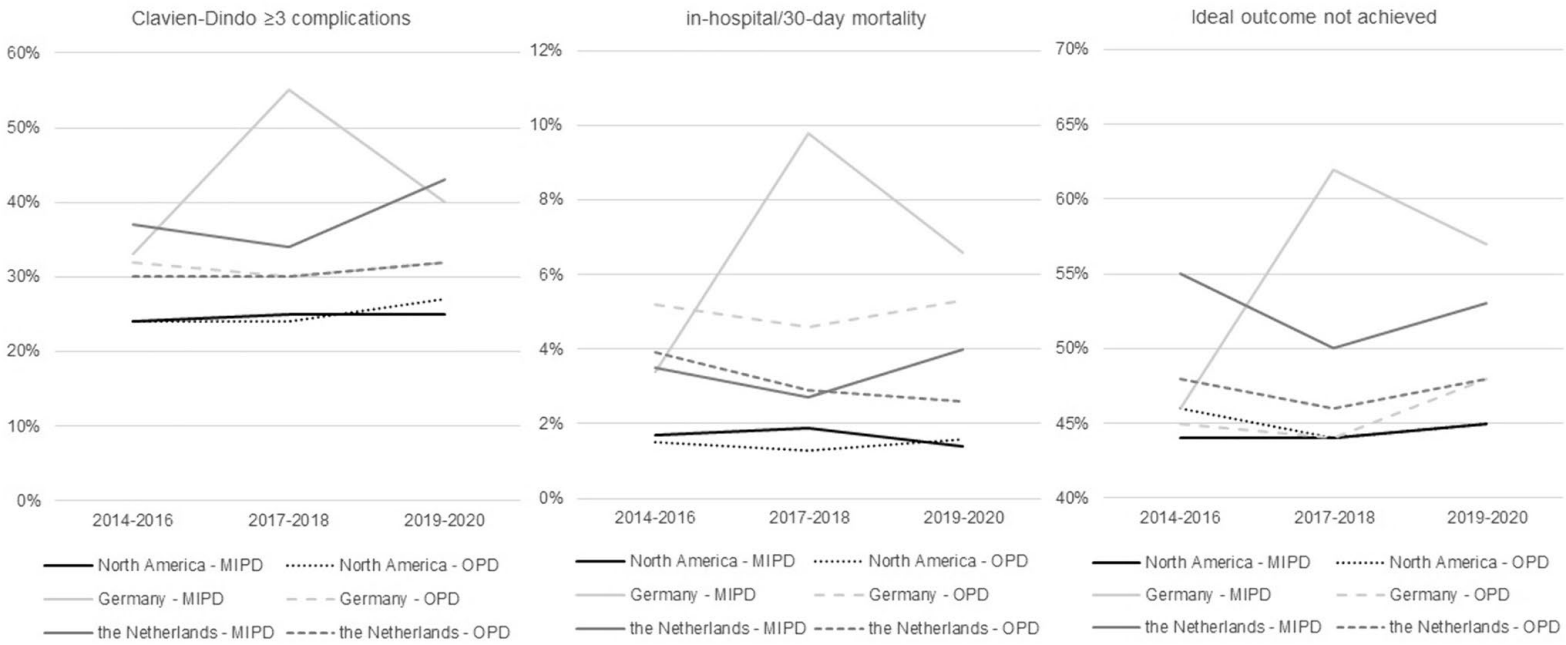
Trend in outcomes after minimally invasive and open pancreatoduodenectomy in three transatlantic audits of pancreatic surgery

Figure 2 shows the rates of major morbidity, mortality, and not achieving Ideal Outcome after MIPD and OPD within North America, Germany, and the Netherlands in three time periods (2014–2020). In North America, complication rates between MIPD and OPD were not significantly different in each time period, unlike Germany and the Netherlands, where worse outcomes for MIPD were found especially in the 2017–2018 time period (Suppl Tables 10–12). In North America, the complication rates after both MIPD and OPD remained stable.

## Discussion

This first transatlantic study focusing on MIPD use and outcome in the four audits from North America, Germany, the Netherlands, and Sweden identified considerable differences in the MIPD use (8%, 4%, 17%, and 0.1%, respectively). Patient characteristics and outcome also differed between the audits for both MIPD and OPD, with best outcomes recorded in North America.

As other international studies assessing the use of MIPD are lacking, our findings cannot be compared to previous studies. How to explain the observed differences between the four audits? At first, the observed differences in use of MIPD among countries could be explained by different implementation strategies. The rapid increase of MIPD use in the Netherlands is clearly related to the two successive training programs for laparoscopic and thereafter robotic pancreatoduodenectomy (LAELAPS-2 and -3), resulting in a standardized and nationwide implementation of MIPD [25, 26]. Whereas North American surgeons and centers implemented MIPD first, no such structured approach was used here, as well as in Germany and Sweden. Consequently, the largest increase in use of MIPD was found in the Netherlands (3% to 23%, *p* < 0.001), compared to North America (7% to 11%, *p* < 0.001) and Germany (1% to 3%, *p* = 0.02). Next, in both the Netherlands and Germany the use of laparoscopic MIPD clearly reduced and nearly stopped, by the concerning results of the LEOPARD-2 randomized trial [27]. This did not apply to North America, were a stable 3% of patients are treated by laparoscopic MIPD over time, with the use of robotic MIPD increasing. A detailed comparison of both MIPD approaches is beyond the scope of the current analysis but a clearly lower conversion rate was seen with robotic MIPD (range 9–37% vs 28–45%), as compared to laparoscopic MIPD.

Within all countries, low-risk patients are more often selected for MIPD. Factors associated with the use of MIPD included no heart failure, better performance status, and no vascular resections (which could reflect less vascular involvement and less advanced tumors). Additionally, patients with malignant diagnosis were less likely to undergo MIPD within all countries, and it is well known that patients with malignancy have less risk factors for morbidity after surgery (i.e. hard pancreas and dilated pancreatic duct) [28]. In line with these results, our study also found that within all audits (and in the Netherlands individually), patients undergoing MIPD had a higher risk of POPF (e.g. smaller tumors, soft pancreas). These findings reflect the selection bias present in the cohort and highlight the need for propensity-score matching and especially randomized trials to compare outcomes between MIPD and OPD. Four randomized trials are available which compared laparoscopic MIPD with OPD [27, 29–31]. Only recently, the first two randomized trials including robotic MIPD are completed (EUROPA [32] and DIPLOMA-2 [33]) both are not published yet.

Within the current study, many differences in baseline characteristics of patients undergoing MIPD between countries were observed. Large differences were observed for BMI > 30 (ALD: 16%), and ASA score (ALD: 48%), see Table 2. BMI is known to be higher in North America, compared to Europe [34]. The difference in ASA scores could be due to registration issues as ASA is known to have a high rate of inter-rater variability, attributing to large differences within countries [35–37]. To enhance adequate comparison between audits, composite outcome such as Ideal Outcome, and highly standardized morbidity such as by the ISGPS are probably most useful [19–21, 38]. GAPASURG will continue to focus on harmonizing the registration of these variables in audits to improve the reliability of comparisons.

Besides these differences in baseline characteristics, the indications for MIPD also differed by country, raising questions regarding the difference in patient selection among countries. This observation In the Netherlands, eligibility criteria for MIPD were determined by both nationwide MIPD training programs [25, 26]; patients without vascular contact, BMI lower than 35 kg/m^2^ and no history of (chronic) pancreatitis. Clearly, as surgeons move through and beyond their learning curve, patient selection criteria will be expanded. In North America, Sweden, and Germany no published nationwide consensus criteria exist on patient selection for MIPD.

The current study also addressed rather concerning nationwide differences in outcomes between MIPD and OPD. Most importantly, none of the three audits demonstrated differences in mortality rates between MIPD and OPD. In Germany, in the period 2017–2018 the mortality difference was the largest (10% MIPD vs 5% OPD). A lower rate of major morbidity and clinically relevant POPF was found in North America, compared to Germany and the Netherlands (ALD: 17% and 14%, respectively). No association between postoperative major morbidity and approach was found in multivariable analysis in North America, while in Germany and the Netherlands higher rates of severe morbidity and lower rates of Ideal Outcome were detected in patients after MIPD. This finding could be explained by the earlier adoption of MIPD in North America, leading to more experienced centers within our study. In the Netherlands, MIPD was also independently associated with higher rates of POPF and major morbidity, potentially due to patient selection, and the implementation of the proactive PORSCH trial postoperative regimen which was implemented simultaneously in the final years and increased the complication detection rate [39]. These findings were not supported by a recently published nationwide cohort study comparing MIPD and OPD outcomes and could therefore be influenced by patient selection [40].

The results of this study should be interpreted in light of several limitations. First, whereas the Dutch (DPCA) and Swedish (SNPPRC) audits are both mandatory, reflecting nationwide outcomes, the German (StudoQ) and North American (NSQIP) are not mandatory. This inevitably leads to some extent of selection and registration bias with a potentially disproportionately greater number of higher-volume centers participating in the non-mandatory registries, hampering a true comparison between countries. The extent of this variation cannot be determined since the annual center volume for pancreatoduodenectomy is unavailable in the NSQIP. Second, of all centers that contributed data to the registries, only a minority of centers included MIPD cases (see Supplementary Table 1). Heterogeneity between these specialized centers and the remaining OPD centers may bias the comparison of MIPD and OPD outcomes. Third, although GAPASURG puts effort in harmonizing the audits, some missing variables or differences in variable definitions remain. For example, some factors, such as blood loss, operating time or hospital volume could not be compared. Third, available data are limited to in-hospital and 30-day outcome measurement, during initial hospital stay, or during readmission within 30 days in the same institution. However, if patients were readmitted to a different institution, the StuDoQ and NSQIP might not capture these data. We are unable to determine the extent to which this affects reported mortality and morbidity rates, as these could potentially be lower than the actual rates. Fourth, due to the large size of the present study, small differences can become statistically significant. Therefore, in addition, ALD and RLD are provided. The major strength of this study is the large size with data from three large nationwide and multicenter audits.

In conclusion, this study showed significant international differences in the adoption, patient selection and postoperative outcomes of MIPD and OPD, between four audits of pancreatic surgery. Most importantly, MIPD was not associated with an increase in mortality, but higher complication rates were found in the European registries. the differences found in patient characteristics and outcome require further research in order to evaluate which patients should be selected for MIPD, and to improve quality of surgical care. Outcome of MIPD should be monitored carefully using audits and the impact of training, or lack thereof, and volume should be addressed in further studies. Ultimately randomized trials will have to determine the true merits of MIPD as compared to OPD.

## Supplementary Information

Below is the link to the electronic supplementary material.

Supplementary file1 (DOCX 13 kb)

Supplementary file2 (DOCX 14 kb)

Supplementary file3 (DOCX 15 kb)

Supplementary file4 (DOCX 13 kb)

Supplementary file5 (DOCX 14 kb)

Supplementary file6 (DOCX 13 kb)

Supplementary file7 (DOCX 14 kb)

Supplementary file8 (DOCX 14 kb)

Supplementary file9 (DOCX 13 kb)

Supplementary file10 (DOCX 13 kb)

Supplementary file11 (DOCX 13 kb)

Supplementary file12 (DOCX 13 kb)

Supplementary file13 (DOCX 15 kb)

Supplementary file14 (DOCX 16 kb)

Supplementary file15 (DOCX 15 kb)
