# Supplementary material for: Transatlantic differences in the use and outcome of minimally invasive pancreatoduodenectomy: an international multi-registry analysis
Source: Surg Endosc. 2024 Sep 28;38(12):7099–111. doi: 10.1007/s00464-024-11161-7 (PMC11615030; doi:10.1007/s00464-024-11161-7)
Supplement: Supplementary file 1 — Supplementary file1 (DOCX 13 kb) [file 464_2024_11161_MOESM1_ESM.docx]

## Supplementary Table 1. Distribution of surgical approaches in four audits of pancreatic surgery

|  | **North America (n=29,107)** | **Germany (n=7,586)** | **The Netherlands (n=4,970)** | **Sweden (n=2,413)** |
| --- | --- | --- | --- | --- |
| **Open pancreatoduodenectomy**  Number of centers | 26,431 (91%)  170 | 7,264 (96%)  67 | 4,041 (83%)  17 | 2,392 (100%)  6 |
| **Minimally invasive pancreatoduodenectomy**  *Of which were converted to open*  Number of centers | 2672 (9%)  *562 (21%)*  NA | 303 (4%)  *110 (36%)*  3 | 839 (17%)  *134 (16%)*  11 | 21 (0%)  NA  1 |
| Laparoscopic pancreatoduodenectomy  *Of which were converted to open* | 812 (3%)  *362 (45%)* | 161 (2%)  *57 (35%)* | 305 (7%)  *84 (28%)* | - |
| Robotic pancreatoduodenectomy  *Of which were converted to open* | 1,331 (5%)  *200 (15%)* | 142 (2%)  *53 (37%)* | 534 (11%)  *50 (9%)* | 21 (0%)  NA |
| Other approaches | 529 (2%) | 0 (0%) | 0 (0%) | - |
| *Missing* | *4 (0%)* | *19 (0%)* | *90 (4%)* | *-* |
