# Supplementary material for: Transatlantic differences in the use and outcome of minimally invasive pancreatoduodenectomy: an international multi-registry analysis
Source: Surg Endosc. 2024 Sep 28;38(12):7099–111. doi: 10.1007/s00464-024-11161-7 (PMC11615030; doi:10.1007/s00464-024-11161-7)
Supplement: Supplementary file 2 — Supplementary file2 (DOCX 14 kb) [file 464_2024_11161_MOESM2_ESM.docx]

## Supplementary Table 2. Predictors for the use of MIPD in the total cohort

|  | **Univariable analysis**  **OR (95% CI)** | **P-**  **value^a^** | **Multivariable analysis**  **OR (95% CI)** | **P-**  **value^b^** |
| --- | --- | --- | --- | --- |
| **Age** | 0.99 (0.99-1.00) | 0.851 |  |  |
| **Female** | 1.01 (0.94-1.08) | 0.786 |  |  |
| **Registry**  North America  Germany  the Netherlands | reference 0.51 (0.46-0.58) 2.56 (2.35-2.79) | **<0.001 <0.001** | reference 0.51 (0.45-0.58) 2.54 (2.32-2.79) | **<0.001 <0.001** |
| **BMI** | 1.00 (0.99-1.01) | **0.105** |  |  |
| **Diabetes** | 0.91 (0.84-0.99) | **0.032** |  |  |
| **COPD** | 1.36 (1.17-1.57) | **<0.001** |  |  |
| **Cardiac heart failure** | 0.53 (0.39-0.71) | **<0.001** | 0.72 (2.32-2.79) | **0.030** |
| **Dialysis** | 2.09 (1.49-2.92) | **<0.001** |  |  |
| **Performance status** Independent  Partially dependent  Fully dependent | reference 0.98 (0.76-1.26) 0.15 (0.02-1.06) | 0.873 **0.057** | reference 0.74 (0.57-0.95) 0.24 (0.03-1.77) | **0.021** 0.14 |
| **ASA score ≥ 3** | 0.76 (0.70-0.81) | **<0.001** |  |  |
| **Pre-operative biliary drainage** No  Yes – ERCP  Yes – PTCD | reference 1.00 (0.93-1.08) 0.94 (0.75-1.19) | 0.945 0.613 |  |  |
| **Operation year** | 1.10 (1.09-1.12) | **<0.001** | 1.12 (1.09-1.14) | **<0.001** |
| **Low risk for POPF** | 0.84 (0.76-0.92) | **<0.001** | 0.84 (0.76-0.91) | **<0.001** |
| **Vascular resection*** No  Vein  Artery  Vein and artery | reference 0.59 (0.52-0.68) 0.88 (0.65-1.19) 0.59 (0.45-0.79) | **<0.001 0.402 <0.001** | reference 0.61 (0.54-0.69) 0.85 (0.63-1.16) 0.65 (0.48-0.86) | **<0.001** 0.309 **0.003** |
| **Histological diagnosis**  Pancreatic adenocarcinoma  Ampullary carcinoma  Distal cholangiocarcinoma  Duodenal carcinoma  Neuroendocrine tumor  IPMN  MCN / serous cystadenoma  Chronic pancreatitis  SPN  Intestinal adenoma  Other | reference  1.77 (1.53-2.05) 1.33 (1.17-1.52) 1.24 (1.02-1.51) 1.45 (1.35-1.68) 2.01 (1.77-2.28) 1.23 (0.93-1.64) 0.73 (0.59-0.89) 1.18 (0.68-2.04) 5.26 (3.58-7.75) 1.14 (0.99-1.29) | **<0.001 <0.001 0.033 <0.001 <0.001 0.143 0.003** 0.558 **<0.001 0.062** | reference 1.41 (1.21-1.64) 1.09 (0.96-1.25) 0.95 (0.77-1.16) 1.32 (1.13-1.53) 1.74 (1.53-1.99) 1.23 (0.92-1.63) 0.81 (0.65-0.99) 1.07 (0.61-1.86) 1.99 (1.34-2.96) 1.07 (0.94-1.23) | **<0.001** 0.175 0.595 **<0.001 <0.001** 0.158 **0.045** 0.816 <0.001 0.309 |
| CI, confidence interval; BMI, body mass index (kg/m^2^); COPD, chronic obstructive pulmonary disease; ASA, American Society of Anesthesiologists physical status classification system; ERCP, endoscopic retrograde cholangio- and pancreaticography; PTCD, percutaneous transhepatic cholagio drainage; POPF, postoperative pancreatic fistula; IPMN, intraductal papillary mucinous neoplasm; MCN, mucinous cystic neoplasm; SPN, solid-pseudopapillary neoplasm. | | | | |
