# Supplementary material for: Transatlantic differences in the use and outcome of minimally invasive pancreatoduodenectomy: an international multi-registry analysis
Source: Surg Endosc. 2024 Sep 28;38(12):7099–111. doi: 10.1007/s00464-024-11161-7 (PMC11615030; doi:10.1007/s00464-024-11161-7)
Supplement: Supplementary file 3 — Supplementary file3 (DOCX 15 kb) [file 464_2024_11161_MOESM3_ESM.docx]

## Supplementary Table 3. Predictors for Clavien Dindo ≥3 morbidity after pancreatoduodenectomy per country

|  | **North America (n=28,573)*** | | **Germany (n=7,557)**** | | | | **The Netherlands (n=4,697)***** | | | |
| --- | --- | --- | --- | --- | --- | --- | --- | --- | --- | --- |
|  | **Univariable analysis**  **OR (95 CI)** | **P-value^a^** | **Univariable analysis**  **OR (95 CI)** | **P-value^a^** | **Multivariable analysis**  **OR (95 CI)** | **P-value^a^** | **Univariable analysis**  **OR (95 CI)** | **P-value^a^** | **Multivariable analysis**  **OR (95 CI)** | **P-value^a^** |
| **Age** | NA | NA | NA | NA | 1.01 (1.00-1.01) | **<0.001** | NA | **NA** | 1.01 (1.00-1.01) | **0.012** |
| **BMI** |  |  |  |  | 1.01 (1.01-1.02) | **0.002** |  |  | 1.00 (0.99-1.01) | 0.349 |
| **Diabetes** |  |  |  |  | 0.82 (0.73-0.92) | **0.001** |  |  | 0.68 (0.57-0.81) | **<0.001** |
| **Cardiac heart failure** |  |  |  |  | 1.03 (0.89-1.20) | 0.667 |  |  | 0.96 (0.66-1.39) | 0.820 |
| **Performance status** Independent  Partially dependent  Fully dependent |  |  |  |  | reference 1.55 (1.23-1.97) 20.86 (9.29-52.49) | **<0.001 <0.001** |  |  | reference 1.04 (0.79-1.35) very large CI | 0.793 0.956 |
| **ASA score ≥ 3** |  |  |  |  | 1.41 (1.27-1.57) | **<0.001** |  |  | 1.23 (1.06-1.42) | **0.008** |
| **Biliary drainage** No  Yes – ERCP  Yes – PTC |  |  |  |  | reference 0.99 (0.89-1.10) NR | 0.877 |  |  | reference 0.79 (0.69-0.91) 0.96 (0.70-1.30) | **0.001** 0.776 |
| **Operation year** |  |  |  |  | 0.99 (0.97-1.02) | 0.741 |  |  | 1.01 (0.98-1.04) | 0.661 |
| **POPF low risk** |  |  |  |  | 0.69 (0.59-0.82) | **<0.001** |  |  | 0.53 (0.45-0.62) | **<0.001** |
| **Vascular resection** |  |  |  |  | 0.99 (0.85-1.16) | 0.949 |  |  | 1.04 (0.87-1.24) | 0.669 |
| **Malignant diagnosis** |  |  |  |  | 0.79 (0.71-0.89) | **<0.001** |  |  | 0.77 (0.66-0.91) | **0.001** |
| **MIPD** | 1.01 (0.91-1.11) | 0.911 | 1.59 (1.26-2.01) | **<0.001** | 1.56 (1.23-1.97) | **<0.001** | 1.43 (1.23-1.67) | **<0.001** | 1.38 (1.17-1.62) | **<0.001** |
| NA: Not applicable. CI, confidence interval; BMI, body mass index (kg/m^2^); ASA, American Society of Anesthesiologists physical status classification system; ERCP, endoscopic retrograde cholangio- and pancreaticography; PTC, percutaneous transhepatic cholangio drainage; POPF, postoperative pancreatic fistula; ^a^Bold numbers indicate statistical significance. *Total exl missing values in univariable analysis: 533 observations deleted due to missing values. **Total exl missing values in multivariable analysis: 28 observations deleted due to missing values. ***Total exl missing values in multivariable analysis: 272 observations deleted due to missing values. | | | | | | | | | | |
