# Supplementary material for: Transatlantic differences in the use and outcome of minimally invasive pancreatoduodenectomy: an international multi-registry analysis
Source: Surg Endosc. 2024 Sep 28;38(12):7099–111. doi: 10.1007/s00464-024-11161-7 (PMC11615030; doi:10.1007/s00464-024-11161-7)
Supplement: Supplementary file 4 — Supplementary file4 (DOCX 13 kb) [file 464_2024_11161_MOESM4_ESM.docx]

## Supplementary Table 4. Predictors for Clavien Dindo ≥3 morbidity after pancreatoduodenectomy in the total cohort

|  | **Total (n=40,477)*** | | |  |
| --- | --- | --- | --- | --- |
|  | **Univariable analysis**  **OR (95 CI)** | **P-value^a^** | **Multivariable analysis**  **OR (95 CI)** | **P-value^a^** |
| **Age** | NA | NA | 1.01 (1.01-1.01) | **<0.001** |
| **BMI** |  |  | 1.01 (1.01-1.01) | **<0.001** |
| **Diabetes** |  |  | 0.82 (0.78-0.87) | **<0.001** |
| **Cardiac heart failure** |  |  | 1.39 (1.23-1.58) | **<0.001** |
| **Performance status** Independent  Partially dependent  Fully dependent |  |  | reference 1.58 (1.37-1.83) 9.06 (5.27-15.6) | **<0.001 <0.001** |
| **ASA score ≥ 3** |  |  | 1.02 (0.97-1.08) | 0.344 |
| **Biliary drainage** No  Yes – ERCP  Yes – PTC |  |  | reference 0.88 (0.84-0.92) 1.06 (0.92-1.22) | **<0.001** 0.396 |
| **Operation year** |  |  | 1.02 (1.01-1.03) | **<0.001** |
| **POPF low risk** |  |  | 0.65 (0.60-0.69) | **<0.001** |
| **Vascular resection** |  |  | 1.16 (1.09-1.23) | **<0.001** |
| **Malignant diagnosis** |  |  | 0.77 (0.73-0.81) | **<0.001** |
| **MIPD** | 1.18 (1.09-1.27) | **<0.001** | 1.16 (1.07-1.26) | **<0.001** |
| NA: Not applicable. CI, confidence interval; BMI, body mass index (kg/m^2^); ASA, American Society of Anesthesiologists physical status classification system; ERCP, endoscopic retrograde cholangio- and pancreaticography; PTC, percutaneous transhepatic cholangio drainage; POPF, postoperative pancreatic fistula; ^a^Bold numbers indicate statistical significance. *Total excl missing values in multivariable analysis: 1,185 observations deleted due to missing values | | | | |
