# Supplementary material for: Transatlantic differences in the use and outcome of minimally invasive pancreatoduodenectomy: an international multi-registry analysis
Source: Surg Endosc. 2024 Sep 28;38(12):7099–111. doi: 10.1007/s00464-024-11161-7 (PMC11615030; doi:10.1007/s00464-024-11161-7)
Supplement: Supplementary file 5 — Supplementary file5 (DOCX 14 kb) [file 464_2024_11161_MOESM5_ESM.docx]

## Supplementary Table 5. Predictors for POPF after pancreatoduodenectomy per audit

|  | **North America (n=28,324)*** | | **Germany (n=7,556)**** | | **The Netherlands (n=4,803)***** | | | |
| --- | --- | --- | --- | --- | --- | --- | --- | --- |
|  | **Univariable analysis**  **OR (95 CI)** | **P-value^a^** | **Univariable analysis**  **OR (95 CI)** | **P-value^a^** | **Univariable analysis**  **OR (95 CI)** | **P-value^a^** | **Multivariable analysis**  **OR (95 CI)** | **P-value^a^** |
| **Age** | NA | NA | NA | NA | NA | NA | 1.01 (0.99-1.01) | 0.077 |
| **BMI** |  |  |  |  |  |  | 1.00 (0.99-1.01) | 0.279 |
| **Diabetes** |  |  |  |  |  |  | 0.66 (0.53-0.83) | **<0.001** |
| **Cardiac heart failure** |  |  |  |  |  |  | 1.12 (0.72-1.75) | 0.614 |
| **Performance status** Independent  Partially dependent  Fully dependent |  |  |  |  |  |  | reference 0.86 (0.59-1.23)  very large CI | 0.416 0.958 |
| **ASA score ≥ 3** |  |  |  |  |  |  | 1.06 (0.89-1.28) | 0.515 |
| **Biliary drainage** No  Yes – ERCP  Yes – PTC |  |  |  |  |  |  | reference 0.83 (0.69-0.98) 0.57 (0.36-0.89) | **0.028 0.015** |
| **Operation year** |  |  |  |  |  |  | 1.08 (1.03-1.12) | **<0.001** |
| **Low risk of POPF** |  |  |  |  |  |  | 0.37 (0.29-0.47) | **<0.001** |
| **Vascular resection** |  |  |  |  |  |  | 0.54 (0.41-0.71) | **<0.001** |
| **Malignant diagnosis** |  |  |  |  |  |  | 0.76 (0.63-0.92) | **0.004** |
| **MIPD** | 0.97 (0.84-1.13) | 0.702 | 1.27 (0.94-1.73) | 0.119 | 1.80 (1.50-2.16) | **<0.001** | 1.54 (1.28-1.87) | **<0.001** |
| NA: Not applicable. CI, confidence interval; BMI, body mass index (kg/m^2^); ASA, American Society of Anesthesiologists physical status classification system; ERCP, endoscopic retrograde cholangio- and pancreaticography; PTC, percutaneous transhepatic cholagio drainage; POPF, postoperative pancreatic fistula; ^a^Bold numbers indicate statistical significance. *Total exl missing values in univariable analysis: 533 observations deleted due to missing values. **Total exl missing values in univariable analysis: 29 observations deleted due to missing values. ***Total exl missing values in multivariable analysis: 166 observations deleted due to missing values. | | | | | | | | |
