# Supplementary material for: Transatlantic differences in the use and outcome of minimally invasive pancreatoduodenectomy: an international multi-registry analysis
Source: Surg Endosc. 2024 Sep 28;38(12):7099–111. doi: 10.1007/s00464-024-11161-7 (PMC11615030; doi:10.1007/s00464-024-11161-7)
Supplement: Supplementary file 6 — Supplementary file6 (DOCX 13 kb) [file 464_2024_11161_MOESM6_ESM.docx]

## Supplementary Table 6. Predictors for POPF after pancreatoduodenectomy in the total cohort

|  | **Total (n=40,351)*** | | |  |
| --- | --- | --- | --- | --- |
|  | **Univariable analysis**  **OR (95 CI)** | **P-value^a^** | **Multivariable analysis**  **OR (95 CI)** | **P-value^a^** |
| **Age** | NA | NA | 1.01 (1.00-1.01) | **<0.001** |
| **BMI** |  |  | 1.01 (1.01-1.02) | **<0.001** |
| **Diabetes** |  |  | 0.72 (0.67-0.78) | **<0.001** |
| **Cardiac heart failure** |  |  | 1.32 (1.12-1.56) | **0.001** |
| **Performance status** Independent  Partially dependent  Fully dependent |  |  | reference 1.46 (1.19-1.77) 3.88 (2.38-6.31) | **<0.001 <0.001** |
| **ASA score ≥ 3** |  |  | 0.83 (0.78-0.89) | **<0.001** |
| **Biliary drainage** No  Yes – ERCP  Yes – PTCD |  |  | reference 0.94 (0.88-1.00) 0.91 (0.73-1.13) | 0.067 0.392 |
| **Operation year** |  |  | 1.12 (1.09-1.14) | **<0.001** |
| **POPF low risk** |  |  | 0.49 (0.44-0.54) | **<0.001** |
| **Vascular resection** |  |  | 0.72 (0.66-0.79) | **<0.001** |
| **Malignant diagnosis** |  |  | 0.64-0.59-0.69) | **<0.001** |
| **MIPD** | 1.28 (1.16-1.42) | **<0.001** | 1.16 (1.05-1.29) | **<0.001** |
| NA: Not applicable. CI, confidence interval; BMI, body mass index (kg/m^2^); ASA, American Society of Anesthesiologists physical status classification system; ERCP, endoscopic retrograde cholangio- and pancreaticography; PTC, percutaneous transhepatic cholagio drainage; POPF, postoperative pancreatic fistula; ^a^Bold numbers indicate statistical significance. *Total exl missing values in multivariable analysis: 1,297 observations deleted due to missing values. | | | | |
