# Supplementary material for: Transatlantic differences in the use and outcome of minimally invasive pancreatoduodenectomy: an international multi-registry analysis
Source: Surg Endosc. 2024 Sep 28;38(12):7099–111. doi: 10.1007/s00464-024-11161-7 (PMC11615030; doi:10.1007/s00464-024-11161-7)
Supplement: Supplementary file 7 — Supplementary file7 (DOCX 14 kb) [file 464_2024_11161_MOESM7_ESM.docx]

## Supplementary Table 7. Predictors for in-hospital/30-day mortality after pancreatoduodenectomy

|  | **North America (n=28,567)*** | | **Germany (n=7,558)**** | | **The Netherlands (n=4,867)***** | | **Total  (n=40,994)****** | |
| --- | --- | --- | --- | --- | --- | --- | --- | --- |
|  | **Univariable analysis**  **OR (95 CI)** | **P-value^a^** | **Univariable analysis**  **OR (95 CI)** | **P-value^a^** | **Univariable analysis**  **OR (95 CI)** | **P-value^a^** | **Multivariable analysis**  **OR (95 CI)** | **P-value^a^** |
| **Age** | NA | NA | NA | NA | NA | **NA** | NA | NA |
| **BMI** |  |  |  |  |  |  |  |  |
| **Diabetes** |  |  |  |  |  |  |  |  |
| **Cardiac heart failure** |  |  |  |  |  |  |  |  |
| **Performance status** Independent  Partially dependent  Fully dependent |  |  |  |  |  |  |  |  |
| **ASA score ≥ 3** |  |  |  |  |  |  |  |  |
| **Biliary drainage** No  Yes – ERCP  Yes – PTCD |  |  |  |  |  |  |  |  |
| **Operation year** |  |  |  |  |  |  |  |  |
| **POPF low risk** |  |  |  |  |  |  |  |  |
| **Vascular resection** |  |  |  |  |  |  |  |  |
| **Malignant diagnosis** |  |  |  |  |  |  |  |  |
| **MIPD** | 1.11 (0.78-1.57) | 0.562 | 1.32 (0.83-2.11) | 0.239 | 1.06 (0.70-1.59) | 0.779 | 1.09 (0.87-1.36) | 0.473 |
| NR: Not Registered. NA: Not applicable. CI, confidence interval; BMI, body mass index (kg/m^2^); ASA, American Society of Anesthesiologists physical status classification system; ERCP, endoscopic retrograde cholangio- and pancreaticography; PTCD, percutaneous transhepatic cholagio drainage; POPF, postoperative pancreatic fistula; ^a^Bold numbers indicate statistical significance. *Total exl missing values in univariable analysis: 539 observations deleted due to missing values. **Total exl missing values in univariable analysis: 27 observations deleted due to missing values. ***Total exl missing values in univariable analysis: 102 observations deleted due to missing values. ****Total exl missing values in univariable analysis: 668 observations deleted due to missing values. | | | | | | | | |
