# Supplementary material for: Transatlantic differences in the use and outcome of minimally invasive pancreatoduodenectomy: an international multi-registry analysis
Source: Surg Endosc. 2024 Sep 28;38(12):7099–111. doi: 10.1007/s00464-024-11161-7 (PMC11615030; doi:10.1007/s00464-024-11161-7)
Supplement: Supplementary file 8 — Supplementary file8 (DOCX 14 kb) [file 464_2024_11161_MOESM8_ESM.docx]

## Supplementary Table 8. Predictors for failing to achieve Ideal outcome per audit

|  | **North America (n=28,401)*** | | **Germany (n=7,520)**** | | | | **The Netherlands (n=4,699)***** | | | |
| --- | --- | --- | --- | --- | --- | --- | --- | --- | --- | --- |
|  | **Univariable analysis**  **OR (95 CI)** | **P-value^a^** | **Univariable analysis**  **OR (95 CI)** | **P-value^a^** | **Multivariable analysis**  **OR (95 CI)** | **P-value^a^** | **Univariable analysis**  **OR (95 CI)** | **P-value^a^** | **Multivariable analysis**  **OR (95 CI)** | **P-value^a^** |
| **Age** | NA | NA | NA | NA | 1.01 (1.01-1.02) | **<0.001** | NA | NA | 1.01 (1.01-1.02) | **<0.001** |
| **BMI** |  |  |  |  | 1.02 (1.01-1.04) | **<0.001** |  |  | 1.00 (0.99-1.01) | 0.301 |
| **Diabetes** |  |  |  |  | 0.87 (0.78-0.97) | **0.011** |  |  | 0.77 (0.65-0.90) | **0.003** |
| **Cardiac heart failure** |  |  |  |  | 1.04 (0.89-1.19) | 0.636 |  |  | 0.85 (0.59-1.21) | 0.357 |
| **Performance status** Independent  Partially dependent  Fully dependent |  |  |  |  | reference 1.81 (1.42-2.30) 28.4 (6.88-117.20) | **<0.001 <0.001** |  |  | reference 1.21 (0.96-1.52)  very large CI | 0.114 0.954 |
| **ASA score ≥ 3** |  |  |  |  | 1.35 (1.23-1.49) | **<0.001** |  |  | 1.25 (1.09-1.44) | 0.002 |
| **Biliary drainage** No  Yes – ERCP  Yes – PTCD |  |  |  |  | reference 0.98 (0.88-1.08) NR | 0.631 |  |  | reference 0.75 (0.65-0.85) 0.88 (0.66-1.18) | **<0.001** 0.384 |
| **Operation year** |  |  |  |  | 1.03 (1.00-1.05) | **0.029** |  |  | 0.99 (0.96-1.02) | 0.433 |
| **POPF low risk** |  |  |  |  | 0.70 (0.60-0.82) | **<0.001** |  |  | 0.58 (0.49-0.67) | **<0.001** |
| **Vascular resection** |  |  |  |  | 0.89 (0.77-1.03) | 0.131 |  |  | 1.02 (0.87-1.20) | 0.799 |
| **Malignant diagnosis** |  |  |  |  | 0.73 (0.65-0.81) | **<0.001** |  |  | 0.76 (0.65-0.87) | **<0.001** |
| **MIPD** | 0.97 (0.89-1.06) | 0.524 | 1.38 (1.09-1.74) | **0.007** | 1.33 (1.05-1.69) | **0.017** | 1.23 (1.06-1.44) | **0.006** | 1.21 (1.03-1.31) | **0.020** |
| NA: Not applicable. CI, confidence interval; BMI, body mass index (kg/m^2^); ASA, American Society of Anesthesiologists physical status classification system; ERCP, endoscopic retrograde cholangio- and pancreaticography; PTCD, percutaneous transhepatic cholagio drainage; POPF, postoperative pancreatic fistula; ^a^Bold numbers indicate statistical significance. *Total exl missing values in univariable analysis: 705 observations deleted due to missing values. **Total exl missing values in multivariable analysis: 62 observations deleted due to missing values. ***Total exl missing values in multivariable analysis: 270 observations deleted due to missing values. | | | | | | | | | | |
