# Supplementary material for: Transatlantic differences in the use and outcome of minimally invasive pancreatoduodenectomy: an international multi-registry analysis
Source: Surg Endosc. 2024 Sep 28;38(12):7099–111. doi: 10.1007/s00464-024-11161-7 (PMC11615030; doi:10.1007/s00464-024-11161-7)
Supplement: Supplementary file 9 — Supplementary file9 (DOCX 13 kb) [file 464_2024_11161_MOESM9_ESM.docx]

## Supplementary Table 9. Predictors for failing to achieve Ideal outcome in the total cohort

|  | **Total (n=40,286)*** | | |  |
| --- | --- | --- | --- | --- |
|  | **Univariable analysis**  **OR (95 CI)** | **P-value^a^** | **Multivariable analysis**  **OR (95 CI)** | **P-value^a^** |
| **Age** | NA | NA | 1.01 (1.01-1.01) | **<0.001** |
| **BMI** |  |  | 1.01 (1.01-1.01) | **<0.001** |
| **Diabetes** |  |  | 0.91 (0.87-0.96) | **<0.001** |
| **Cardiac heart failure** |  |  | 1.13 (1.01-1.28) | **0.038** |
| **Performance status** Independent  Partially dependent  Fully dependent |  |  | reference 1.71 (1.49-1.96) 12.85 (5.57-29.64) | **<0.001 <0.001** |
| **ASA score ≥ 3** |  |  | 1.16 (1.11-1.21) | **<0.001** |
| **Biliary drainage** No  Yes – ERCP  Yes – PTCD |  |  | reference 0.82 (0.79-0.86) 1.12 (0.99-1.27) | **<0.001** 0.075 |
| **Operation year** |  |  | 0.99 (0.99-1.01) | 0.547 |
| **POPF low risk** |  |  | 0.75 (0.71-0.79) | **<0.001** |
| **Vascular resection** |  |  | 1.17 (1.11-1.24) | **<0.001** |
| **Malignant diagnosis** |  |  | 0.80 (0.76-0.84) | **<0.001** |
| **MIPD** | 1.08 (1.00-1.16) | **0.044** | 1.08 (1.01-1.16) | **0.034** |
| NA: Not applicable. CI, confidence interval; BMI, body mass index (kg/m^2^); ASA, American Society of Anesthesiologists physical status classification system; ERCP, endoscopic retrograde cholangio- and pancreaticography; PTCD, percutaneous transhepatic cholagio drainage; POPF, postoperative pancreatic fistula; ^a^Bold numbers indicate statistical significance. *Total exl missing values in multivariable analysis: 1,376 observations deleted due to missing values | | | | |
