# Supplementary material for: Transatlantic differences in the use and outcome of minimally invasive pancreatoduodenectomy: an international multi-registry analysis
Source: Surg Endosc. 2024 Sep 28;38(12):7099–111. doi: 10.1007/s00464-024-11161-7 (PMC11615030; doi:10.1007/s00464-024-11161-7)
Supplement: Supplementary file 10 — Supplementary file10 (DOCX 13 kb) [file 464_2024_11161_MOESM10_ESM.docx]

## Supplementary Table 10. Complication rates over time NSQIP

|  | **2014-2016** | | | **2017-2018** | | | **2019-2020** | | |
| --- | --- | --- | --- | --- | --- | --- | --- | --- | --- |
|  | **MIPD  (n = 692)** | **OPD (n= 10,102)** | **ALD** | **MIPD (n = 591)** | **OPD (n = 7,886)** | **ALD** | **MIPD (n = 860)** | **OPD (n = 8,443)** | **ALD** |
| Clavien-Dindo ≥3 | 167 (24%) | 2,384 (24%) | 0.0% | 146 (25%) | 1,911 (24%) | 1.0% | 219 (25%) | 2,238 (27%) | 2.0% |
| POPF | 43 (6.3%) | 780 (7.8%) | 2.5% | 49 (8.3%) | 737 (9.4%) | 0.9% | 120 (14%) | 1,168 (14%) | 0.0% |
| Not achieving Ideal Outcome | 303 (44%) | 4,588 (46%) | 2.0% | 3,463 (44%) | 261 (44%) | 0.0% | 380 (45%) | 3,798 (45%) | 0.0% |
| Mortality | 12 (1.7%) | 151 (1.5%) | 0.2% | 11 (1.9%) | 103 (1.3%) | 0.6% | 12 (1.4%) | 136 (1.6%) | 0.2% |

Bold numbers indicate statistical significance
