# Supplementary material for: Transatlantic differences in the use and outcome of minimally invasive pancreatoduodenectomy: an international multi-registry analysis
Source: Surg Endosc. 2024 Sep 28;38(12):7099–111. doi: 10.1007/s00464-024-11161-7 (PMC11615030; doi:10.1007/s00464-024-11161-7)
Supplement: Supplementary file 11 — Supplementary file11 (DOCX 13 kb) [file 464_2024_11161_MOESM11_ESM.docx]

## Supplementary Table 11. Complication rates over time STUDOQ

|  | **2014-2016** | | | **2017-2018** | | | **2019-2020** | | |
| --- | --- | --- | --- | --- | --- | --- | --- | --- | --- |
|  | **MIPD  (n = 119)** | **OPD (n= 2,366)** | **ALD** | **MIPD (n = 92)** | **OPD (n = 2,292)** | **ALD** | **MIPD (n = 92)** | **OPD (n = 2,606)** | **ALD** |
| Clavien-Dindo ≥3 | 39 (33%) | 757 (32%) | 1.0% | 51 (55%) | 678 (30%) | **25.0%** | 37 (40%) | 830 (32%) | 8.0% |
| POPF | 18 (15%) | 349 (15%) | 0.0% | 20 (22%) | 274 (12%) | **10.0%** | 14 (15%) | 391 (15%) | 0.0% |
| Not achieving Ideal Outcome | 54 (46%) | 1,065 (45%) | 1.0% | 56 (62%) | 1,005 (44%) | **18.0%** | 52 (57%) | 1,253 (48%) | 9.0% |
| Mortality | 4 (3.4%) | 123 (5.2%) | 1.8% | 9 (9.8%) | 106 (4.6%) | **5.2%** | 7 (6.6%) | 139 (5.3%) | 1.3% |

Bold numbers indicate statistical significance
