# Supplementary material for: Transatlantic differences in the use and outcome of minimally invasive pancreatoduodenectomy: an international multi-registry analysis
Source: Surg Endosc. 2024 Sep 28;38(12):7099–111. doi: 10.1007/s00464-024-11161-7 (PMC11615030; doi:10.1007/s00464-024-11161-7)
Supplement: Supplementary file 12 — Supplementary file12 (DOCX 13 kb) [file 464_2024_11161_MOESM12_ESM.docx]

## Supplementary Table 12. Complication rates over time DPCA

|  | **2014-2016** | | | **2017-2018** | | | **2019-2020** | | |
| --- | --- | --- | --- | --- | --- | --- | --- | --- | --- |
|  | **MIPD  (n = 226)** | **OPD (n= 1,787)** | **ALD** | **MIPD (n = 259)** | **OPD (n = 1,172)** | **ALD** | **MIPD (n = 354)** | **OPD (n = 1,082)** | **ALD** |
| Clavien-Dindo ≥3 | 81 (37%) | 519 (30%) | **7.0%** | 86 (34%) | 341 (30%) | 4% | 149 (43%) | 331 (32%) | **9.0%** |
| POPF | 59 (26%) | 219 (12%) | **14.0%** | 49 (19%) | 186 (16%) | 3% | 93 (26%) | 197 (18%) | **8.0%** |
| Not achieving Ideal Outcome | 120 (55%) | 822 (48%) | **7.0%** | 127 (50%) | 528 (46%) | 4.0% | 184 (53%) | 508 (48%) | 5.0% |
| Mortality | 8 (3.5%) | 70 (3.9%) | 0.4% | 7 (2.7%) | 34 (2.9%) | 0.2% | 14 (4.0%) | 28 (2.6%) | 1.4% |

Bold numbers indicate statistical significance
