# Supplementary material for: Transatlantic differences in the use and outcome of minimally invasive pancreatoduodenectomy: an international multi-registry analysis
Source: Surg Endosc. 2024 Sep 28;38(12):7099–111. doi: 10.1007/s00464-024-11161-7 (PMC11615030; doi:10.1007/s00464-024-11161-7)
Supplement: Supplementary file 13 — Supplementary file13 (DOCX 15 kb) [file 464_2024_11161_MOESM13_ESM.docx]

## Supplementary Table 13. Baseline characteristics and operative outcomes of patients after pancreatoduodenectomy in four transatlantic audits (including missing)

|  | **North America**  **(n=29,107)** | **Germany (n=7,586)** | **The Netherlands (n=4,970)** | **Sweden (n=2,413)** | **Total (n=44,076)** |
| --- | --- | --- | --- | --- | --- |
| **Age**, median (IQR) *Missing* | 66.0 (58.0-73.0) *37* | 69.0 (60.0-76.0) *0* | 68.0 (61.0-74.0) *8* | 70.0 (63.2-75.0) *187* | 67.0 (59.0-74.0) *232* |
| **Female** *Missing* | 13,534 (46%) *0* | 3,267 (43%) *3* | 2,210 (45%) *4* | 1,152 (48%) *0* | 20,163 (46%) *7* |
| **BMI**, median (IQR) *Missing* | 26.5 (23.3-30.4) *155* | 24.9 (22.5-27.8) *28* | 24.8 (22.4-27.6) *156* | 24.9 (22.5-27.8) *91* | 25.8 (23.0-29.5) *430* |
| **Diabetes**  *Missing* | 7,714 (27%) *0* | 1,928 (25%) *8* | 1,009 (25%) *945* | 497 (21%) *19* | 11,148 (25%) *972* |
| **COPD**  *Missing* | 1,179 (4%) *0* | 371 (5%) *8* | 549 (14%) *945* | 130 (6%) *31* | 2,229 (5%) *984* |
| **Heart failure**  *Missing* | 114 (0.4%) *0* | 936 (13%) *153* | 123 (3%) *945* | 640 (27%) *29* | 1,813 (4%) *1,127* |
| **Dialysis**  *Missing* | 92 (0.3%) *0* | 23 (0.3%) *9* | 163 (4%) *945* | 9 (4%) *2,174* | 287 (1%) *3,128* |
| **Performance status** Independent  Partially dependent  Fully dependent *Missing* | 28,825 (99%) 226 (1%) 20 (<0.1%) *36* | 7,199 (95%) 310 (4%) 57 (1%) *20* | 3,905 (91%) 373 (9%) 1 (<0.1%) *691* | 2,204 (93%) 157 (7%) 1 (<0.1%) *51* | 42,133 (97%) 1,066 (3%) 79 (0.2%) *798* |
| **ASA score ≥ 3** *Missing* | 23,113 (79%) *19* | 3,894 (51%) *3* | 1,355 (29%) *72* | 669 (28%) *20* | 29,031 (66%) *1,127* |
| **Preoperative biliary drainage** No  Yes – ERCP  Yes – PTCD*   *Missing* | 12,783 (46%) 14,191 (51%) 859 (3%) *1,274* | 4,740 (63%) 2,831 (37%) NR *15* | 2,204 (46%) 2,330 (49%) 226 (5%) *210* | 1,009 (42%) 1,384 (58%) NR *20* | 20,736 (49%) 20,736 (49%) 1,085 (3%)* *1519* |
| **Neoadjuvant chemotherapy^#^**  *Missing* | 5,621 (34%) *78* | 230 (6%) *7* | 342 (16%) *87* | 68 (6%) | 6,261 (26%) *174* |
| **Histological diagnosis**  Pancreatic adenocarcinoma  Ampullary carcinoma  Distal cholangiocarcinoma  Duodenal carcinoma  Neuroendocrine tumor  IPMN  MCN / serous cystadenoma  Chronic pancreatitis  SPN  Intestinal adenoma  Other  *Missing* | 16,795 (59%) 843 (3%) 2,189 (8%) 879 (3%) 1,793 (6%) 1,779 (6%) 503 (2%) 1,079 (4%) 127 (0.4%) 0 (0%) 1,630 (9%) *490* | 3,813 (51%) 603 (8%) 631 (8%) 199 (3%) 279 (4%) 434 (6%) 126 (2%) 740 (10%) 34 (1%) 0 (0%) 663 (9%) *64* | 2,064 (42%) 634 (13%) 656 (13%) 313 (6%) 236 (5%) 364 (7%) 41 (1%) 139 (3%) 20 (0.4%) 130 (3%) 312 (6%) *61* | 1,133 (51%) 247 (11%) 295 (13%) 157 (7%) 42 (2%) 21 (1%) 0 (0%) 67 (3%) 0 (0%) 61 (3%) 194 (9%) *196* | 23,805 (55%) 2,327 (5%) 3,771 (9%) 1,548 (4%) 2,350 (5%) 2,598 (6%) 670 (2%) 2,025 (5%) 181 (0.4%) 191 (0.4%) 3,799 (9%) *811* |
| **Type of PD**  Pylorus preserving PD  Pylorus resecting PD / classic Whipple | 10,456 (36%) 18,651 (64%) | 5,433 (72%) 2,153 (28%) | 2,546 (51%) 2,424 (49%) | 581 (24%) 1,832 (76%) | 19,016 (43%) 25,060 (57%) |
| **MIPD**  *Missing* | 2,143 (8%) *533* | 303 (4%) *19* | 839 (17%) *90* | 21 (0.1%) *388* | 3,285 (8%) *3,055* |
| **Vascular resection**  No  Vein  Artery  Vein and artery  *Missing* | 23,505 (82%) 3,697 (12%) 581 (2%) 961 (3%) *363* | 6,659 (88%) 900 (12%) 21 (0.3%) 6 (<0.1%) *19* | 4,141 (84%) 696 (14%) 54 (1%) 20 (0.4%) *59* | 1,968 (82%) 426 (18%) 8 (0.3%) 8 (0.3%0 3 | 36,273 (83%) 5,719 (13%) 664 (2%) 995 (2%) *425* |
| Numbers are depicted as N(%) unless indicated otherwise. MIPD: minimally invasive pancreatoduodenectomy. OPD: open pancreatoduodenectomy. NR: not registered. ALD: absolute largest difference. RLD: relative largest difference ^#^Comparing patients undergoing MIS surgery among the GAPASUG countries. *Used as a surrogate for pre-operative vascular involvement. ^$^Only comparing vascular resection (yes or no), as groups become too small to compare. | | | | | |
