# Supplementary material for: Transatlantic differences in the use and outcome of minimally invasive pancreatoduodenectomy: an international multi-registry analysis
Source: Surg Endosc. 2024 Sep 28;38(12):7099–111. doi: 10.1007/s00464-024-11161-7 (PMC11615030; doi:10.1007/s00464-024-11161-7)
Supplement: Supplementary file 14 — Supplementary file14 (DOCX 16 kb) [file 464_2024_11161_MOESM14_ESM.docx]

## Supplementary Table 14. Characteristics of patients after MIPD and OPD in three transatlantic audits (including missing)

|  | **North America** | | **P-value** | **Germany** | | **P-value** | **The Netherlands** | | **P-value** | **MIS GAPASURG^#^** | | |
| --- | --- | --- | --- | --- | --- | --- | --- | --- | --- | --- | --- | --- |
|  | **MIPD** (n=2,143) | **OPD** (n=26,431) |  | **MIPD** (n=303) | **OPD** (n=7,264) |  | **MIPD** (n=839) | **OPD** (n=4,041) |  | **ALD** | **RLD** | **P-value** |
| **Age**, median (IQR) *Missing* | 66.0 (58.0-73.0) *0* | 66.0 (58.0-73.0) *37* | 0.237 | 69.0 (58.5-76.0) *0* | 69.0 (60.0-76.0) *0* | 0.353 | 69.0 (61.0-75.0) *2* | 68.0 (60.0-74.0) *6* | **0.010** | 3.0 | 1.0 | **<0.001** |
| **Female** *Missing* | 1,011 (47%) *0* | 12,277 (46%) *0* | 0.516 | 134 (44%) *0* | 3,123 (43%) *3* | 0.676 | 361 (43%) *2* | 1,809 (45%) *2* | 0.380 | 4% | 1.1 | 0.114 |
| **BMI**, median (IQR) *Missing* | 26.9 (23.7-30.9) *5* | 26.5 (23.3-30.3) *148* | **<0.001** | 25.5 (23.1-28.3) *0* | 24.9 (22.5-27.8) *25* | **0.030** | 25.1 (22.6-27.7) *25* | 24.7 (22.3-27.5) *181* | **0.065** | 1.8 | 1.1 | **<0.001** |
| **BMI ≥ 30 kg/m^2^** | 610 (29%) | 6,962 (26%) | **0.040** | 46 (15%) | 1,048 (14%) | 0.733 | 106 (13%) | 501 (13%) | 0.865 | 16% | 2.2 | **<0.001** |
| **Diabetes** | 541 (25%) | 7,031 (27%) | 0.171 | 65 (21%) | 1,859 (26%) | 0.104 | 172 (24%) | 820 (25%) | 0.307 | 4% | 1.2 | **<0.001** |
| **COPD** | 88 (4%) | 1,079 (4%) | 0.957 | 15 (5%) | 356 (5%) | 0.970 | 114 (16%) | 424 (13%) | 0.075 | 12% | 4.0 | 0.288 |
| **Cardiac heart failure** | 8 (0.4%) | 106 (0.4%) | 0.845 | 16 (5%) | 918 (13%) | **<0.001** | 28 (4%) | 94 (3%) | 0.191 | 4.6% | 12.5 | **<0.001** |
| **Dialysis** | 6 (0.3%) | 83 (0.3%) | 0.786 | 1 (0.3%) | 22 (0.3%) | 0.610 | 36 (5%) | 126 (4%) | 0.203 | 4.7% | 4.7 | **<0.001** |
| **Performance status** Independent  Partially dependent  Fully dependent *Missing* | 2,129 (99%) 13 (1%) 0 (0%) *1* | 26,168 (99%) 209 (1%) 20 (<0.1%) *34* | 0.427 | 298 (98%) 4 (1%) 1 (0.3%) *0* | 6,889 (95%) 305 (4%) 56 (1%) *14* | **0.020** | 672 (93%) 48 (7%) 0 (0%) *118* | 3,150 (91%) 322 (9%) 1 (<0.1%) *568* | **0.046** | 6% 6% 1% | 1.1 7.0 NA | **<0.001** |
| **ASA score ≥ 3** *Missing* | 1,637 (77%) *5* | 21,071 (80%) *13* | **<0.001** | 167 (55%) *0* | 3,716 (51%) *2* | 0.178 | 234 (29%) *18* | 1,102 (28%) *52* | 0.610 | 48% | 2.7 | **<0.001** |
| **Biliary drainage** No  Yes – ERCP  Yes – PTCD   *Missing* | 999 (48%) 1,029 (50%) 46 (2%) *69* | 11,555 (46%) 12,919 (51%) 800 (3%) *1,157* | **0.012** | 216 (71%) 88 (29%) NR *0* | 4,519 (62%) 2,739 (38%) NR *6* | **0.002** | 354 (44%) 423 (52%) 35 (4%) *24* | 1,814 (47%) 1,859 (48%) 186 (5%) *182* | 0.150 | 27% | 1.6 | **<0.001** |
| **High risk for POPF**   *Missing* | 1,591 (79%) *133* | 18,429 (76%) *2,169* | **0.001** | 221 (85%) *42* | 4,930 (80%) *1,092* | 0.057 | 575 (79%) *113* | 2,423 (73%) *719* | **<0.001** | 6% | 1.1 | 0.108 |
| **Vascular resection*** No  Vein  Artery  Vein and artery *Missing* | 1,846 (87%) 180 (9%) 42 (2%) 48 (2%) *27* | 21,231 (81%) 3,464 (13%) 524 (2%) 887 (3%) *325* | **<0.001** | 266 (88%) 36 (12%) 1 (0.3%) 0 (0%) *0* | 6,374 (88%) 864 (12%) 20 (0.3%) 6 (<0.1%) *0* | **<0.001** | 769 (92%) 58 (7%) 5 (1%) 2 (0.2%) *5* | 3,303 (83%) 626 (16%) 47 (1%) 18 (1%) *47* | **<0.001** | 5% 5% 2% 2% | 1.1 1.7 6.7 NA | **<0.001^$^** |
| **Malignant disease** *Missing* | 1,442 (69%) *40* | 18,909 (73%) *435* | **<0.001** | 178 (59%) *1* | 5,055 (70%) *60* | **<0.001** | 585 (74%) | 3,013 (77%) | **0.023** | 15% | 1.3 | **<0.001** |
| Numbers are depicted as N(%) unless indicated otherwise. MIPD: minimally invasive pancreatoduodenectomy. OPD: open pancreatoduodenectomy. NR: not registered. ALD: absolute largest difference. RLD: relative largest difference ^#^Comparing patients undergoing MIS surgery among the GAPASUG countries. *Used as a surrogate for pre-operative vascular involvement. ^$^Only comparing vascular resection (yes or no), as groups become too small to compare. | | | | | | | | | | | | |
