# Supplementary material for: Transatlantic differences in the use and outcome of minimally invasive pancreatoduodenectomy: an international multi-registry analysis
Source: Surg Endosc. 2024 Sep 28;38(12):7099–111. doi: 10.1007/s00464-024-11161-7 (PMC11615030; doi:10.1007/s00464-024-11161-7)
Supplement: Supplementary file 15 — Supplementary file15 (DOCX 15 kb) [file 464_2024_11161_MOESM15_ESM.docx]

## Supplementary Table 15. Outcomes after MIPD and OPD in GAPASURG and three transatlantic audits (including missing)

|  | **North America** | | **P-value** | **Germany** | | **P-value** | **The Netherlands** | | **P-value** | **MIS GAPASURG^#^** | | |
| --- | --- | --- | --- | --- | --- | --- | --- | --- | --- | --- | --- | --- |
|  | **MIPD** (n=2,143) | **OPD**(n=26,431) |  | **MIPD** (n=303) | **OPD** (n=7,264) |  | **MIPD** (n=839) | **OPD** (n=4,041) |  | **ALD** | **RLD** | **P-value** |
| Safety outcomes | | | | | | | | | | | | |
| **Clavien-Dindo ≥ 3** *Missing* | 532 (25%) *0* | 6,533 (25%) *0* | 0.911 | 127 (42%) *0* | 2,265 (31%) *9* | **<0.001** | 316 (38%) *17* | 1,191 (30%) *118* | **<0.001** | 17% | 1.7 | **<0.001** |
| **Radiologic intervention** *Missing* | 299 (14%) *0* | 3,629 (19%) *0* | 0.770 | NR | NR | NA | 236 (29%) *15* | 769 (20%) *108* | **<0.001** | 15% | 2.1 | **<0.001** |
| **Reoperation** *Missing* | 145 (7%) *0* | 1,459 (6%) *0* | **0.016** | 77 (25%) *1* | 1,198 (17%) *47* | **<0.001** | 87 (11%) *18* | 306 (8%) *128* | **0.009** | 18% | 3.6 | **<0.001** |
| **Unplanned ICU admission** *Missing* | NR | NR | NA | 46 (15%) *0* | 776 (11%) *9* | **0.014** | 115 (14%) *8* | 412 (10%) *63* | **0.003** | 1% | 1.1 | 0.566 |
| **In-hospital/30-day mortality** *Missing* | 35 (2%) *0* | 390 (2%) *6* | 0.563 | 20 (7%) *0* | 368 (5%) *8* | 0.237 | 29 (4%) *2* | 132 (3%) *10* | 0.780 | 5% | 7.2 | **<0.001** |
| **Ideal Outcome not achieved** *Missing* | 944 (44%) *16* | 11,849 (45%) *169* | 0.524 | 162 (54%) *3* | 3,323 (46%) *43* | **0.007** | 431 (53%) *19* | 1,858 (47%) *113* | **0.006** | 13% | 1.3 | **<0.001** |
| Other morbidity | | | | | | | | | | | | |
| **POPF grade B/C** *Missing* | 212 (10%) *20* | 2,685 (10%) *229* | 0.702 | 52 (17%) *0* | 1,014 (14%) *10* | 0.119 | 201 (24%) *4* | 602 (15%) *21* | **<0.001** | 14% | 2.4 | **<0.001** |
| **Bile leakage grade B/C** *Missing* | NR | NR | NA | 27 (9%) *0* | 378 (5%) *0* | **0.005** | 74 (9%) *6* | 197 (5%) *50* | **<0.001** | 0% | NA | 0.989 |
| **DGE grade B/C** *Missing* | 358 (17%) *20* | 4,407 (17%) *270* | 0.986 | 33 (11%) *0* | 731 (10%) *11* | 0.646 | 74 (9%) *6* | 751 (5%) *50* | **<0.001** | 2% | 1.2 | **<0.001** |
| **PPH grade B/C** *Missing* | NR | NR | NA | 49 (16%) *0* | 665 (9%) *11* | **<0.001** | 106 (13%) *10* | 295 (7%) *49* | **<0.001** | 3% | 1.2 | 0.142 |
| **Pneumonia** *Missing* | 66 (3%) | 1,059 (4%) | **0.034** | 33 (11%) *0* | 434 (6%) *10* | **<0.001** | 45 (6%) *93* | 158 (5%) *866* | 0.242 | 8% | 3.7 | **<0.001** |
| **Surgical site infection** *Missing* | 158 (7%) | 2,562 (10%) | **<0.001** | 39 (13%) *9* | 834 (12%) *154* | 0.424 | 51 (7%) *99* | 338 (11%) *870* | **0.002** | 6% | 1.9 | **0.001** |
| Other outcomes | | | | | | | | | | | | |
| **LOS <75 percentile** *Missing* | 411 (20%) | 6,467 (25%) | **<0.001** | 77 (26%) *4* | 1,738 (24%) *35* | 0.498 | 201 (24%) *14* | 936 (23%) *51* | 0.577 | 14% | 2.2 | **<0.001** |
| **Readmission** *Missing* | 427 (20%) *0* | 4,462 (17%) *10* | **<0.001** | 33 (11%) *1* | 624 (9%) *51* | 0.170 | 149 (19%) *43* | 678 (18%) *188* | 0.451 | 9% | 1.8 | **<0.001** |
| ICU, intensive care unit; POPF, postoperative pancreatic fistula; DGE, delayed gastric emptying; PPH, post-pancreatectomy hemorrhage; LOS, length of hospital stay in days. | | | | | | | | | | | | |
